# Modeling rainfall-induced 2D inundation simulation based on the ANN-derived models with precipitation and water-level measurements at roadside IoT sensors

**DOI:** 10.1038/s41598-023-44276-3

**Published:** 2023-10-17

**Authors:** Shiang-Jen Wu

**Affiliations:** Department of Civil and Disaster Prevention Engineering, National United University, Miaoli, 360302 Taiwan

**Keywords:** Hydrology, Natural hazards

## Abstract

This study aims to develop a smart model for carrying out two-dimensional (2D) inundation simulation by estimating the gridded inundation depths via the ANN-derived models (ANN_GA-SA_MTF), named SM_EID_2D model. Within the SM_EID_2D model, the rainfall-induced inundation depths at the IoT sensors (i.e., IOT-based grids) are first estimated to be then used in the estimation of inundation depths at the ungauged grids (VIOT-based grids), the resulting flood extents and spatial distribution of inundation of what could be achieved. To facilitate the reliability of the proposed SM_EID_2D model in the 2D inundation simulation, a considerable number of rainfall-induced flood events are generated as the training datasets by coupling the hydrodynamic numerical model (SOBEK) with the simulated gridded rainstorms. To proceed with the model validation and application, the Miaoli City of North Taiwan is selected as the study area, and the associated hydrological and geographical data are adopted in the generation of the training datasets. The results from the model validation indicate that the proposed SM_EID_2D model could provide the gridded inundation-depth hydrographs with a low bias (about 0.02 m) and a high fitness to the validated data (nearly 0.7); also, the spatial distribution of inundated and non-inundated grids as well as the induced flooding extent provided could be well emulated by the proposed SM_EID_2D model under acceptable reliability (0.7). The proposed SM_EID_2D model is also advantageous for the 2D inundation simulation in the real-time delineated subbasins by assembling the emulated inundation depths at the specific grids.

## Introduction

Recently, rainfall-induced floods have severely damaged people’s lives and property, actions attributed to climate change, and extreme rainstorm events^1–4^. Forecasting inundation events triggered by rainstorms plays an essential role in issuing information on the prevention and mitigation of flood-induced hazards, which is helpful for the flood early warning operation. In the past, inundation forecasts could be made by the data-derived approaches based on the precipitation estimations/forecasts and observed water level/runoff with the desired potential flood maps^5–7^. For example, Mustel et al.^6^ presented a data-derived flood-crest forecast algorithm based on the real-time measurements of the runoff velocities at the specific gauges along the river; thus, the resulting short-term forecast information on the peak discharge and its arrival time could be quantified in advance under the known real-time runoff measurements. However, the real-time practical flood-induced runoff and inundation depths, especially in urban areas, might be challenging to measure due to the limitation of measurement equipment, hindrances in data acquisition, accuracy in the parameters of the numerical model for processing and analysis, and spatial uncertainties in the digital elevation map. The above deprivation probably causes uncertainties in the delineated flooding zones and area^4,8–13^. Nevertheless, enhancing the computation power and capability, a group of numerical simulation models based on the rainfall-runoff analysis and flood dynamics routing are comprehensively applied in the 1D/2D inundation/flood simulation^9–11^. For instance, Brandt^10^ proposed a stochastically-based algorithm for delineating the flood risk map by coupling the hydraulic numerical model (HEC-RAS) with consideration of the uncertainties in the boundaries and slopes shown in the digital elevation map (DEM).

Generally speaking, by employing the hydrodynamic numerical models, the at-site inundation depths and induced potential flooding zones, as well as the associated area, can be estimated under consideration of the various types of rainfalls, such as the design rainfall events of the different return periods and the precipitation forecasts as well as observations^13–18^; for example, Ming et al.^16^ developed a 2D hydrodynamic simulation module to perform real-time flooding forecast with the precipitation forecasts provided via the weather numerical models. Although the hydrodynamic numerical models can reproduce the inundation depths at the specific locations and induced flooding areas in the catchment and urban spaces, their simulation performance and accuracy significantly positively rely on the resolutions of the resulting flood-related variates in time and space^10,19^. Additionally, the hydrological and topographical data on high spatial and temporal resolution are more likely to cause complicated routing processes with more parameters requiring calibration and expensive computation time^20^.

AI-created models are comprehensively employed in the inundation simulation to overcome the above resolution-induced disadvantage of the 2D flood simulation. Of these AI-created models, the artificial neural network (ANN) and convolution neural network (CNN) are mainly adopted for numerically describing the nonlinear mathematic relationships with all possible model inputs; these consist of the linear multi-layer network via the multiple training algorithm^21–30^. Accordingly, the AI-created inundation simulation modules could be grouped into region-based (e.g., CNN) and at-site models (e.g., ANN). As for the region-based AI-created CNN model, the 2D flood simulation could be carried out by recognizing the potentially inundated regions in advance, whereby the associated inundation depths could then be obtained^21,24,31^; for example, Yan et al.^31^ proposed a 2D CNN-derived inundation simulation model to predict the water depths at specific locations along the river; the model training was conducted via the machine learning technique. The flooding region should be drawn based on the estimated inundation depths for the gridded-based ANN models. Its extent could be calculated based on the number of grids with nonzero inundation depths at all grids concerned, which are multiplied by the grid size^4,23,25,29,30^; for instance, Wu et al.^4^ proposed an ANN-derived model whose the optimal parameters could be achieved via the modified genetic algorithm, to estimate the ungauged inundation depths with the sent water levels real-time measured and sent at the sensors through the internet of thing (IoT), which are named IoT sensors, to ensemble a flooding zone.

Despite the CNN-related models applying to the 2D flood forecast with the precipitation estimations and forecasts of high resolution in time and space, in theory, the resulting inundation zone from the CNN-based models is mainly recognized from the pooling layers, including the existing flood-related images database and excluding the changes in the required hydraulic and hydrological input factors (e.g., the precipitation and rainfall-induced surface runoff); this might barely efficiently respond to the practical variation and geopolitical limitations regarding the detailed rainfall-runoff-inundation characteristics in time and space^24,32,33^. Contrarily, the ANN-derived model has an excellent algorithm with which to establish the linear and nonlinear relationships between a variety of input–output combinations with good quality of training datasets^18,23^; however, the ANN-derived models could mainly provide the at-site water levels with the rainfall observations and forecasts which hardly applies to emulate information of high resolution in space, such as the time-varying delineation of the inundation zones^23,34^.

The ANN-derived models could successfully and effectively estimate the at-site hydrological factors, but it is hardly applied in the simulation of regional variables in a two-dimensional domain. Therefore, to efficiently carry out the 2D inundation simulation based on the ANN-derived model with the at-site hydrological observations, this study aims to develop a smart model for carrying out 2D inundation simulation by estimating the inundation depths at all grids in a region under given rainfall measurements and forecasts and observed water levels at the roadside IoT sensors during the rainstorms; the estimated inundation depths at all grid could result in the flooding extent. The proposed SM_EID_2D model is anticipated to realistically reproduce the gridded rainfall-induced inundation depths with high accuracy and emulate the corresponding flooding extent and spatial pattern with high reliability, which is advantageous to flood early warning and mitigation.

## Methodology

### Model concept

The purpose of the proposed ANN-derived SM_EID_2D model is to carry out a 2D inundation simulation attributed to the rainstorms in which the inundation depths at all grids and induced flood areas can be quantified. In theory, before developing the AI-created models, a training dataset including a sizeable number of model outputs simulated via the physically-based numerical modeling with numerous model inputs. Thus, in this study, a considerable number of rainfall-induced flood events would be reproduced as the training datasets via the hydrodynamic numerical model with the generated rainstorms within the desired potential flooding regions. Among a group of hydrodynamic numerical models are frequency applied in the flooding simulation, the SOBEK model^35^ can describe the rainfall-runoff-inundation process in the watershed associated with a variety of hydraulic structures (e.g., river channels, bridges, drainage systems, and storage-related infrastructures; thereby, the SOBEK model is adopted in the simulations of the rainfall-induced inundation to estimate the inundation depths at all grids within the study area.

**Figure 1:**
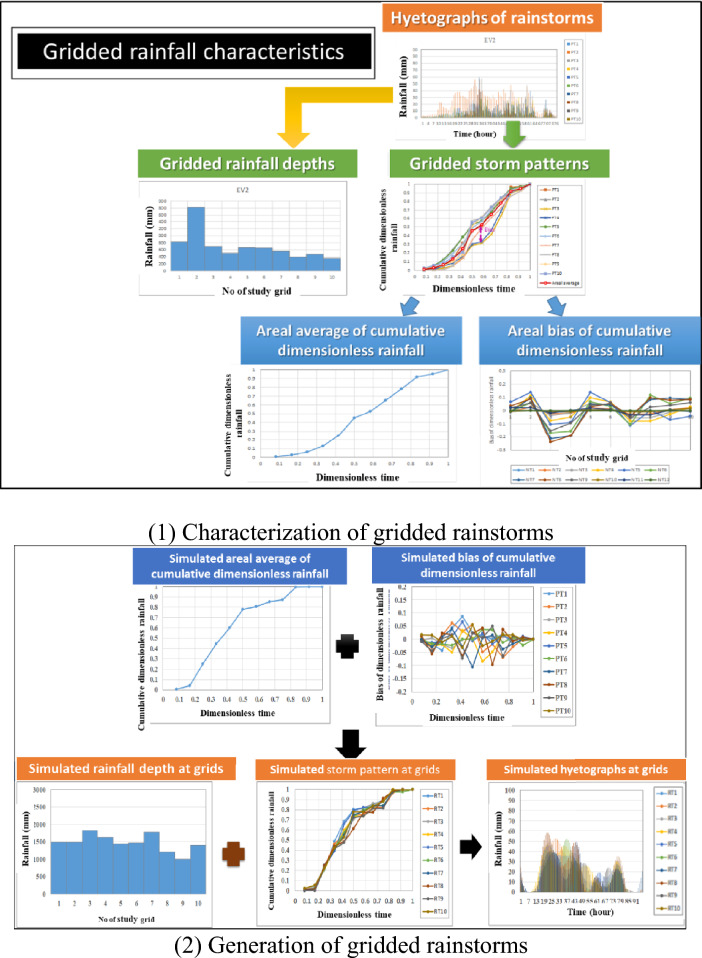
Schematic process of characterizing and generating event-based rainstorms^13,36,37^.

Accordingly, the proposed SM_EID_2D model is developed by coupling two relationships for estimating the inundation depths at IOT-based and VIOT-based grids, established based on ANN-derived models, respectively. Within the proposed SM_EID_2D model, the above two inundation-depth estimation relationships are configured based on a modified ANN model by considering the sensitivity of the model outputs to the model inputs, ANN_GA-SA_MTF^36^. In detail, the ANN-derived relationships at the IOT-based grids are mainly used to estimate the inundation depths at the grids with the IoT sensors caused by the rainstorms, and the other ANN-derived relationships at the VIOT-based grids are employed to emulate the inundation depths at the ungauged grids with the gridded inundation ones at the IOT-based grids. By so doing, the flood region could then be delineated to compute the corresponding inundation extent. Figure 1 shows the schematic process of the 2D inundation simulation to obtain the gridded inundation depths and resulting flooding area. Also, to boost the accuracy of the model outputs, the proposed SM_EID_2D model would be coupled with the real-time error correction algorithm for the estimated water stages in the 2D domain subject to the gauged observations, RTEC_2DIS model^18^; accordingly, the resulting gridded inundation depths at the available grids, including the IOT-based and VIOT-based grids could be adjusted based on the real-time measurements of the inundation depths at the IOT-based grids. Ultimately, the model validation could compare the gridded inundation depths and the corresponding flood extents estimated by the proposed SM_EID_2D model with those from the training datasets comprised of the simulated rainfall-induced flood events.

In total, the development of the proposed SM_EID_2D model could be classified into five components: (1) generation of training datasets including the rainfall-induced inundation events; (2) derivation of the ANN-derived model for the estimation of the inundation depths at the IoT-based grids; (3) training of the ANN-derived model for the estimation of the inundation depths at the VIOT-based grids; (5) adjustment of the inundation-depth estimates at all available grids; and (5) comparison in the gridded inundation-depth and resulting flooding-area hydrographs.

### Simulations of training datasets, including rainfall-induced inundations

While training the AI-created models, a sizable dataset is needed to enhance the reliability of the calibrated parameters^4,13,38,39^. Therefore, a considerable number of simulation cases, including rainfall-induced floods, should be achieved in advance via the hydrodynamic numerical model with the generated rainstorm events. Specifically, to carry out 2D inundation simulation, the rainstorms at all grids (named gridded rainstorms) in the study area should be generated as the facing data for the hydrodynamic numerical model. By so doing, in this study, to train the ANN-derived model (ANN_GA-SA_MTF model), the stochastically-based model for generating the rain fields, the SM_GSTR model^37^, is utilized to simulate the gridded rainstorms with the spatial and temporal statistical properties of the gridded rainfall characteristics. The main concept of the SM_GSTR model is briefly introduced as follows:

Within the SM_GSTR model, the event-based rainstorm events could be characterized into three components: the event-based rainfall durations, gridded rainfall depths, and gridded storm patterns, which are treated as the spatial and spatiotemporal correlated variates, respectively. Specifically, the gridded storm pattern could be classified into two components: the areal average of the dimensionless rainfalls and the associated deviations at the various dimensionless times. In summary, the gridded rainstorm characteristics include the rainfall duration, gridded rainfall depth, areal average of the nondimensional rainfall, and gridded deviation; the process of characterizing and generating the gridded rainstorms into the five gridded rainfall characteristics could be referred to in Fig. 1.

**Figure 2:**
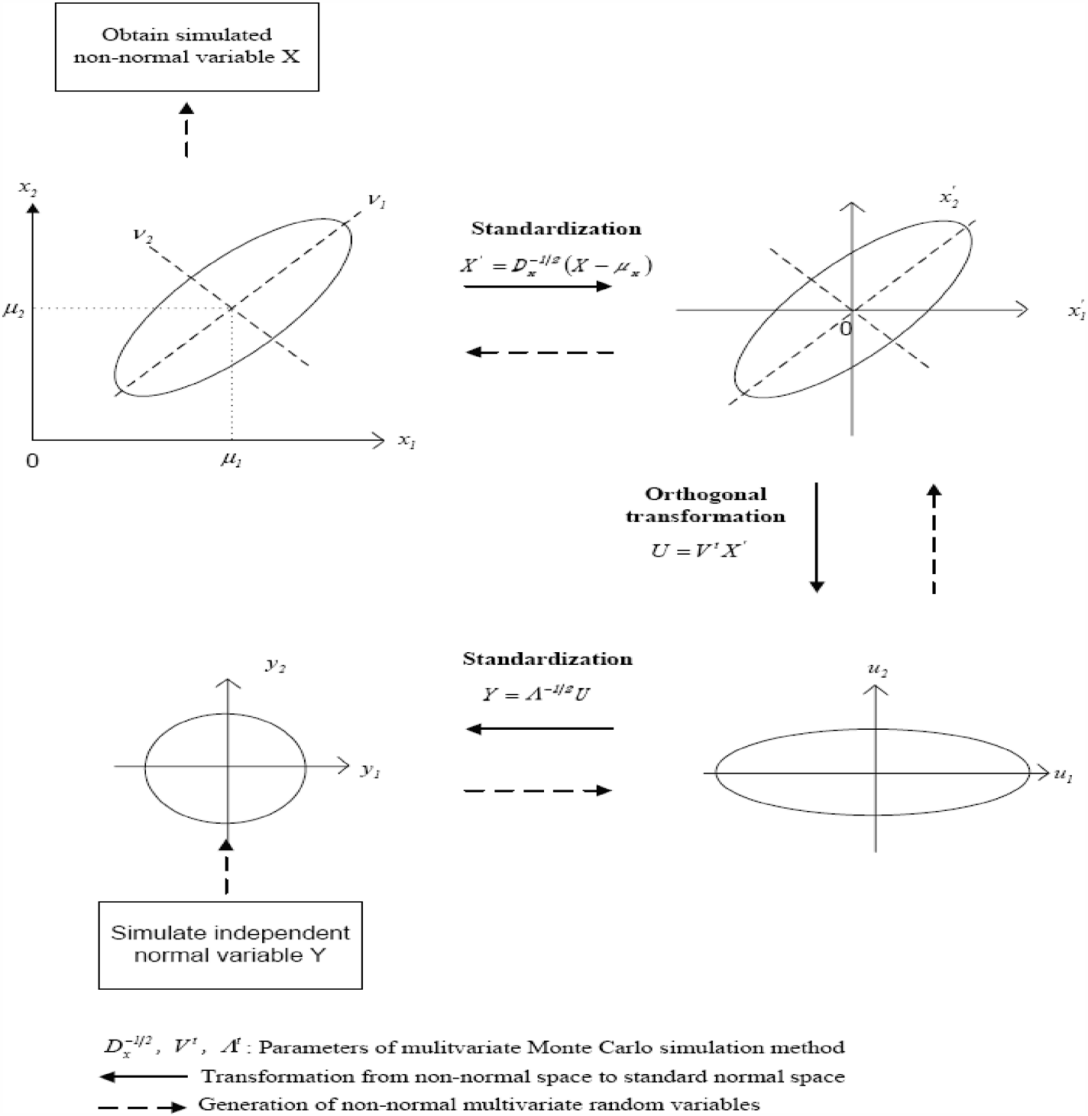
Graphical framework of the normal transformation regarding the correlated multivariate Monte Carlo simulation^41^.

After obtaining the gridded rainfall characteristics, their uncertainties in time and space would be quantified in terms of the statistical properties, including the first four statistical moments, appropriate probability distribution, and correlation coefficients in time and space. As a result of the gridded rainfall characteristics being spatial and temporal variables, the Monte Carlo simulation approach for the correlated and non-normal multivariates^40^, named the MMCS method, is employed to reproduce a considerable number of the gridded rainfall characteristics. Note that the MMCS approach could proceed with three transformation operations: normal, orthogonal, and inverse transformations (see Fig. 2).

In detail, the corresponding standard normal variables to the gridded rainfall characteristic are reproduced in advance and then transferred into the magnitude in the real space through the following equations (Nataf^42^):

1 $$\rho _{{ij}} = \mathop \smallint \limits_{{ - \infty }}^{\infty } \mathop \smallint \limits_{{ - \infty }}^{\infty } \left\lfloor {\frac{{x_{i} - \mu _{i} }}{{\sigma _{i} }}} \right\rfloor \left\lfloor {\frac{{x_{j} - \mu _{j} }}{{\sigma _{j} }}} \right\rfloor \emptyset _{{ij}} \left( {\left\langle {z_{i} ,z_{j} |\rho _{{ij}}^{*} } \right\rangle } \right)dz_{i} dz_{j}$$

2 $$z_{i} = \frac{{x_{i} - \mu_{i} }}{{\sigma_{i} }};\;z_{j} = \frac{{x_{j} - \mu_{j} }}{{\sigma_{j} }}$$

where *X*_*i*_ and *X*_*j*_ are the correlated variables at points *i* and *j*, respectively, with the means ${\mu }_{i}$ and ${\mu }_{j}$, the standard deviations ${\sigma }_{i}$ and ${\sigma }_{j}$, and the correlation coefficient ${\rho }_{ij}$; _*i*_ and *Z*_*j*_ are corresponding bivariate standard normal variables to the variable *X*_*i*_ and *X*_*j*_ with the correlation coefficient $\rho_{ij}^{ * }$ and the joint standard normal density function *ϕ*_*ij*_(∙); eventually, the simulations of the gridded rainfall characteristics are combined as the hyetographs regarding the gridded rainstorms as shown in Fig. 1;

The accuracy and reliability of the resulting gridded rainstorm events in a domain with a high spatial resolution of $1.5\mathrm{ km}\times 1.5\mathrm{ km}$ from the SM_GSTR model have been verified in comparison to the statistical properties of the gridded rainfall characteristics calculated from the historical and simulated data; the corresponding results indicate that the simulated gridded rainfall characteristics are more likely to capture the inherent statistical properties of the observed rainfall characteristics in time and space under consideration of the spatial and temporal correlations; the remaining detailed development and demonstration of the SM_GSTR model successfully applied in the flood simulation could be referred to the investigation by Wu et al.^4,13^.

Afterwards, the SOBEK model is commonly and widely configured for the 2D inundation simulation in watersheds and drainage regions with various hydraulic structures, such as storage-based facilities, drainage-related hydraulic structures, and waterproofing systems^35^. Therefore, in this study, the training datasets for the proposed SM_EID_2D model, including a significant number of simulated rainfall-induced flood events, could be obtained via the SOBEK with the gridded rainstorm simulations.

### Configuration of the SM_EID_2D model

As mentioned earlier, while developing the proposed SM_EID_2D model, the gridded ANN-derived models are utilized in the derivation of the relationships between the rainfall and gauged inundation depths as well as the ungauged ones for the IOT-based and VIOT-based grids; That is to say, the above relationships are established via the modified ANN-based model (i.e., ANN_GA-SA_MTF model). Therefore, in this section, the concept of the ANN_GA-SA_MTF model is briefly introduced; the detailed derivation of the resulting relationships for estimating the inundation depths at the IOT-based and VIOT-based grids from the ANN_GA-SA_MTF model are addressed as follows:

#### Introduction to the ANN-derived model

According to the framework mentioned above of the model development, the proposed SM_EID_2D model is mainly configured by coupling two relationships based on the ANN-derived models used in the estimation of inundation depths at the IOT-based and VIOT-based grids. Therefore, the concept of the ANN_GA-SA_MTF model is briefly expressed as:

Concerning the impact of the variation in the selection of activation functions (see Table 1) and induced uncertainties in the model parameters on the model outputs, Wu et al.^36^ proposed an ANN-derived model, ANN_GA-SA_MTF model, the training of which is conducted via the modified genetic algorithm based on the sensitivity of the model outputs to the model inputs (i.e., GA-SA algorithm)^43^.

**Table 1:** List of the well-known activation functions required in the ANN-derive model^13,36,37^.

Transfer function		Formula	Derivative
TF1	Logistic (soft step, Sigmoid)	$\mathrm{f}\left(\mathrm{x}\right)=\frac{1}{1+{e}^{-\propto x}}$	${\mathrm{f}}^{\mathrm{^{\prime}}}\left(\mathrm{x}\right)=\mathrm{f}(\mathrm{x})(1-\mathrm{f}\left(\mathrm{x}\right))$
TF2	Tcomprisinganh	$\mathrm{f}\left(\mathrm{x}\right)=\mathrm{tanh}\left(\mathrm{x}\right)=\frac{2}{1+{e}^{-2\propto x}}-1$	${\mathrm{f}}^{\mathrm{^{\prime}}}\left(\mathrm{x}\right)=1-{f(x)}^{2}$
TF3	Arctan	$\mathrm{f}\left(\mathrm{x}\right)={tan}^{-1}(\propto x)$	${\mathrm{f}}^{\mathrm{^{\prime}}}\left(\mathrm{x}\right)=\frac{1}{{(\propto x)}^{2}+1}$
TF4	Identity	f(x) = $\propto$ x	f^' (x) = $\propto$
TF5	Rectified linear unit (ReLU)	$${\text{f}}\left( {\text{x}} \right) = \left\{ {\begin{array}{*{20}c} 0 & {for\;x < 0} \\ 1 & {for\;x \ge 0} \\ \end{array} } \right.$$	$${\text{f}}^{\prime } \left( {\text{x}} \right) = \left\{ {\begin{array}{*{20}c} 0 & {for\;x < 0} \\ 1 & {for\;x \ge 0} \\ \end{array} } \right.$$
TF6	Parameteric rectified linear unit (PReLU, leaky ReLU)	$${\text{f}}\left( {\text{x}} \right) = \left\{ {\begin{array}{*{20}c} { \propto x} & {for\;x < 0} \\ x & {for\;x \ge 0} \\ \end{array} } \right.$$	$${\text{f}}^{\prime } \left( {\text{x}} \right) = \left\{ {\begin{array}{*{20}c} \propto & {for\;x < 0} \\ 1 & {for\;x \ge 0} \\ \end{array} } \right.$$
TF7	Exponential linear unit(ELU)	$${\text{f}}\left( {\text{x}} \right) = \left\{ {\begin{array}{*{20}l} { \propto \left( {e^{x} - 1} \right)} \hfill & {for\;x < 0} \hfill \\ x \hfill & {for{\mkern 1mu} x \ge 0} \hfill \\ \end{array} } \right.$$	$${\text{f}}^{\prime } \left( {\text{x}} \right) = \left\{ {\begin{array}{*{20}l} {f\left( x \right) + \propto } \hfill & {for\;x < 0} \hfill \\ 1 \hfill & {for\;x \ge 0} \hfill \\ \end{array} } \right.$$
TF8	Inverse abs (IA)	$\mathrm{y}(\mathrm{x})\frac{x}{1+|\propto x|}$	${\mathrm{y}}^{\mathrm{^{\prime}}}\left(\mathrm{a}\right)=\frac{1}{\left(1+\left|a\propto x\right|\right)^2}$
TF9	Rootsig (RS)	$\mathrm{y}\left(\mathrm{x}\right)=\frac{\propto x}{1+\sqrt{1+{(\propto x)}^{2}}}$	$\mathrm{y{\prime}}\left(\mathrm{x}\right)=\frac{1}{(1+\sqrt{1+{(\propto x)}^{2}})\sqrt{1+{a(\propto x)}^{2}}}$
TF10	Sech function (SF)	$\mathrm{y}\left(\mathrm{x}\right)=\frac{2}{\mathrm{exp}\left(\propto x\right)+\mathrm{exp}(-\propto x)}$	${\mathrm{y}}^{\mathrm{^{\prime}}}\left(\mathrm{x}\right)=-\mathrm{y}\left(\mathrm{x}\right)\mathrm{tanh}(\propto x)$

In detail, while training the ANN_GA-SA_MTF model, a group of associated parameters could be calibrated using the GA-SA algorithm with various activation functions. The corresponding model estimates to the calibrated parameters for a specific activation function could then be obtained; eventually, the weighted average of the resulting model estimates from the various activation functions are defined as the final model outputs through the following equation:

$${\widehat{Y}}_{WA}=\sum_{i=1}^{{N}_{TF}}\left[{W}_{TF}^{i}\times \widehat{Y}\left({\theta }_{TF}^{i}\right)\right]$$

3 $$W_{TF}^{i} = \frac{{\frac{1}{{E\left( {\theta_{TF}^{i} } \right)}}}}{{\mathop \sum \nolimits_{i = 1}^{{N_{TF} }} \frac{1}{{E\left( {\theta_{TF}^{i} } \right)}}}}$$

where $N_{TF}$ denotes the number of transfer functions considered; ${Y}_{k} and {\widehat{Y}}_{k}\left(({\theta }_{TF}^{i}\right)$ account for the observed model inputs and estimated model outputs, respectively, by the ANN_GA-SA_MTF model with the calibrated parameters ${\theta }_{TF}^{i}$ from the ith activation function; and ${W}_{TF}^{i}$ serves as the weighted factor of the ith activation function, calculated from the $E\left({\theta }_{TF}^{i}\right)$ being the objective-function value as below:

4 $$E({\theta }_{TF}^{i})=\sqrt{\frac{1}{{N}_{data}}\sum_{\mathrm{k}=1}^{{N}_{data}}{[{Y}_{k}-{\widehat{Y}}_{k}\left(({\theta }_{TF}^{i}\right)]}^{2}}$$

where ${N}_{data}$ stands for the number of observed hydrological estimates. In this study, within the ANN_GA-SA_MTF model, a neural network with the three neurons located in a hidden layer is configured in the proposed SM_EID_2D model to estimate the inundation depths at the IOT-based and VIOT-based grids.

#### Derivation of relationships for estimating rainfall-induced inundation depths

It is well known that the spatial and temporal correlations should exist in the gridded inundation depths and rainfall^13^; accordingly, for the model training and execution, the spatial and temporal resolutions in the resulting rainfall in the inundation should be identified in advance. To quantify the optimal resolutions in time and space, Wu et al.^13^ carried out the sensitivity analysis for the gridded inundation depths, which were induced by the rainstorms in a rain field of 1.5-km spatial resolution within a potentially inundated zone (spatial resolution of 40 m) via the standard normal equation; consequently, the 3 h and 3 km are treated as the critical resolutions in time and space, respectively.

Therefore, according to the model concept of the proposed SM_EID_2D model addressed in Section "Model concept", the ANN-GA-SA_MTF model is configured for establishing the relationship of the estimated inundation depths at the IOT-based grids with the corresponding areal average rainfall. Note that as for the corresponding areal average rainfall to the IOT-based grids, extracted from the training datasets, including the simulated rainfall-induced inundation simulations, the areal average rainfalls at the current and forward time steps of 3 h are achieved as:

5 $${\overline{R} }_{IOT}^{t}=\frac{1}{{N}_{Rgrid}}\sum_{i=1}^{{N}_{Rgrid}}{R}_{i}^{t}$$

where ${\overline{R} }_{IOT}^{t}$ is the areal-average rainfall at the IOT-based grids for the time step t-hour; ${N}_{Rgrid}$ stands for the number of the rain grids, whose distances are less than the critical resolution of 3 km to the target VIOT-based grid; and ${R}_{i}^{t}$ serves as the rainfalls at the time step t-hour for the *ith* rain grid selected within the distance of 3 km.

Therefore, within the proposed SM_EID_2D model, regarding the estimation of the inundation depths at the IOT-based grids, the resulting relationship between the inundation depth of 1-h lead time with the corresponding areal average rainfalls at the l-hour lead time and forward 3 h as well as the observed inundation depths at the forward 3 h could be established via the ANN_GA-SA_MTF as

6 $${\widehat{h}}_{IOT}^{t+1}={\mathrm{f}}_{{\mathrm{ANN}}_{\mathrm{GA}}-{\mathrm{SA}}_{\mathrm{MTF}}}({\overline{R} }_{IOT}^{t+1},{\overline{R} }_{IOT}^{t},{\overline{R} }_{IOT}^{t-1},{\overline{R} }_{IOT}^{t-2},{h}_{IOT}^{t},{{h}_{IOT}^{t-1},h}_{IOT}^{t-2})$$

where ${\widehat{h}}_{IOT}^{t+1}$ and ${\overline{R} }_{IOT}^{t+1}$ stand for the inundation-depth estimate and areal average rainfall for the lead time (t + 1 h), respectively; and ${(\overline{R} }_{IOT}^{t},{\overline{R} }_{IOT}^{t-1},,{\overline{R} }_{IOT}^{t-2}$ and $({\overline{R} }_{IOT}^{t-2},{h}_{IOT}^{t},{{h}_{IOT}^{t-1},h}_{IOT}^{t-2})$ denote the observed areal average rainfalls and observed inundation depths at the forward three time steps, respectively. As a result, while carrying out the 2D inundation simulation, the inundation depths of 1-h lead time at the IoT sensors could be obtained using Eq. (6) adapted in the proposed SM_EID_2D model in the case of the 3-h observations of the area average rainfall as well as inundation depths and 1-h precipitation forecast given.

#### Derivation of relationship for estimating ungauged inundation depths

In general, the inundation depths are well-known spatial and temporal variables^4,22,44^; thus, the 2D flood simulation should be carried out subject to the corrections of the inundation depths in time and space. The aforementioned relationships of estimating inundation depth as the IOT-based grids could persist in the temporal correlation of the gridded rainfall-induced inundation depths. Hence, to continue the correlation of the inundation depths in space, the ANN-created relationships of the estimated inundation depths between the IOT-based grids and the VIOT-based grids should be established within the proposed SM_EID_2D model.

In detail, with the proposed SM_EID_2D model estimated at the IOT-based grids, the resulting inundation depths are used to emulate the water levels at the VIOT-based grids via the ANN-derived relationships of the inundation depths between the IOT-based and VIOT-based grids. Bo so doing, for training the relationships mentioned above to reflect the spatial correlation in the inundation depths, the spatial average of the inundation depths at the VIOT-based are first estimated by the inverse distance method with the results from the IOT-based grids (named IDW-based estimated inundation depth) as:

7 $$\overline{h}_{IDW, VIOT}^{t} = \mathop \sum \limits_{i = 1}^{{N_{IOT} }} \left[ {h_{IOTi}^{t} \times \left( {\frac{{\frac{1}{{L_{i} }}}}{{\mathop \sum \nolimits_{i}^{3} \frac{1}{{L_{i} }}}}} \right)} \right]$$

in which $h_{IOTi}^{t}$ stands for the estimated inundation depths at the *ith* IOT-based grids, the distance of which to the VIOT-based grid is ${L}_{i}$; and ${N}_{IOT}$ serves as the number of the IOT-based grids with a distance of ${L}_{i}$ to the specific VIOT-based grid. Since the above IDW-related areal average of the hydrological variable is more likely to cause a significant bias^45–47^; thus, an ANN-derived (ANN_GA-SA_MTF) model is developed to adjust the IDW-based estimated inundation depths at the specific VIOT grid (${\overline{h} }_{IDW, VIOT}^{t}$) as:

8 $${{\widehat{h}}_{EST,VIOT}^{t}={f}_{ANN\_GA-SA\_MTF}(\overline{h} }_{IDW,VIOT}^{t})$$

Eventually, the resulting inundation zones could be delineated by assembling the estimated inundation depth at all available, i.e., the inundated and non-inundated grids.

#### Estimation of inundation depths via the proposed SM_EID_2D model

According to the above introduction to the proposed SM_EID_2D model, the inundation depths at the gauges (i.e., IOT-based grids) and ungauged locations (i.e., VIOT-based grids) could be estimated using Eqs. (5) and (8) under the conditions of model inputs, including the observed areal average rainfalls of 3 h and the precipitation forecasts of 1-h lead time as well as the observed water levels of 3 h at the IOT-based grids. As a result, the estimated inundation depths at all available grids could be assembled with the digital elevation map (DEM) to be the flooding zones, the extent of what could be accordingly quantified,

In detail, by carrying out 2D inundation simulation via the proposed SM_EID_2D model, the corresponding areal average of the rainfalls at the forward three hours to the IOT-based grids could be first calculated via Eq. (5); after that, the inundation depths of 1-h lead time at the IOT-based grids could be then achieved via Eq. (6) using the above corresponding areal average rainfall and observed water levels at the IOT-based grids. Accordingly, the spatial average of the inundation depths at the target VIOT-based grids could be computed through Eq. (7), in which the weighs of the inundation depth at the IOT-based grids is subject to the inverse of the distance between the target VIOT-based grid and nearby IOT-based grids. Eventually, the inundation-depth estimates of 1-h lead time at the VIOT-based grids could be emulated via Eq. (8).

**Figure 3:**
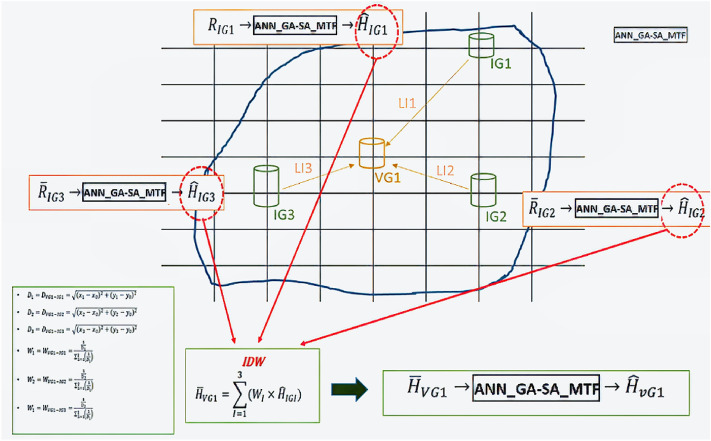
Schematic illustration of proceeding with the 2D inundation simulation via the proposed SM_EID_2D model.

**Figure 4:**
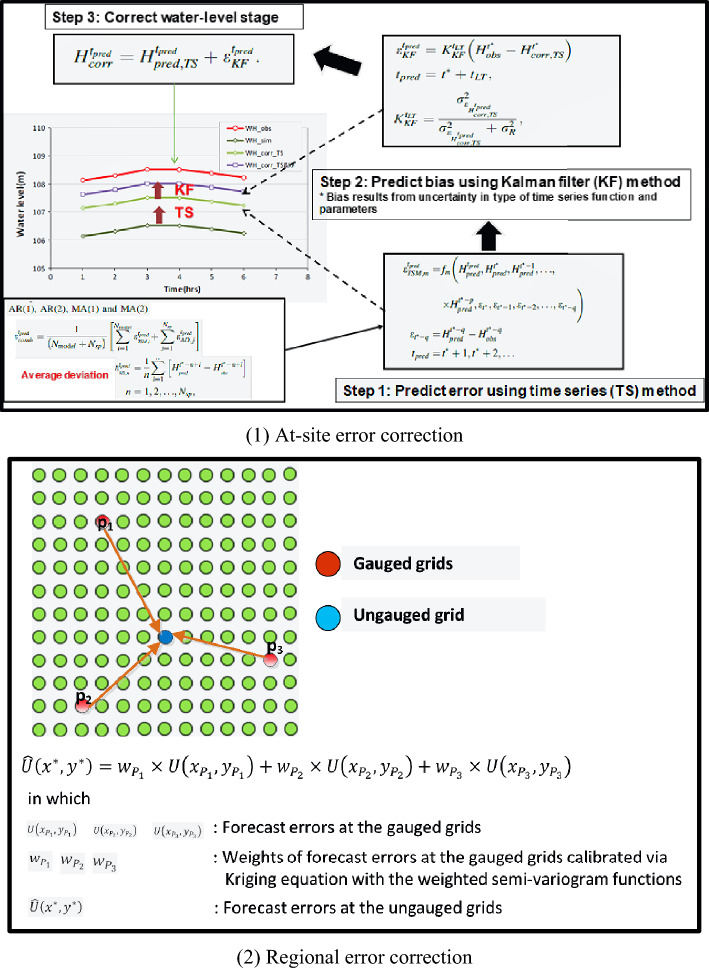
Graphical process of the real-time error correction for 2D inundation simulation via the RTEC_2DIS model^4^.

Figure 4 shows the schematic illustration of proceeding with the 2D inundation simulation via the proposed SM_EID_2D model. In Fig. 3, The gridded inundation depth at the lead time could be estimated via the proposed SM_EID_2D model in the case of a VIOT-based grid (VG1) with three IOT-based grids (i.e., IG1, IG2, and IG3), in which R_IG1_, R_IG2_ and R_IG3_ are treated as the areal average rainfall at the IG1, IG2 and IG3.

### Real-time adjustment of inundation-depth estimates

**Figure 5:**
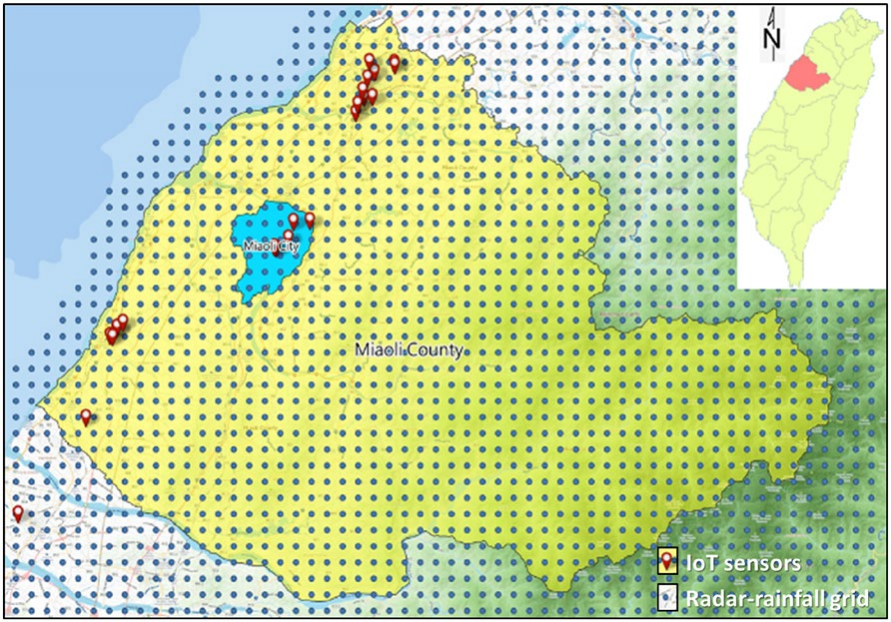
Locations of the study area Miaoli City (symbolled as a blue zone) and associated hydrological measurement gages^4^; this figure is created using QGIS, URL: https://www.qis.com.

Theoretically, the estimated inundation depths at the IOT-based and VIOT-based grids could be reasonably provided using two ANN_GA-SA_MTF models, respectively, coupled with the proposed SM_EID_2D model. Nonetheless, their reliabilities and the accuracies of the results from the AI-created models are commonly impacted due to their dynamic nature, variations of real-world problems, uncertainties in the quality/size of the training datasets, and model parameters^4,13,27,48^. Therefore, to boost the model performance of the proposed SM_EID_2D model, a statistically-based real-time error model algorithm for the 2D inundation simulation (RTEC_2DIS)^18^ (is adopted to adjust the estimated inundation depths at all grids subject to the real-time measurements at the IoT sensors^4,13^; Fig. 5 presents the schematic process of correcting the gauged and ungauged water level estimates in the 2D inundation simulation via the RTEC_2DIS model; the relevant concepts are addressed as follows:

While proceeding with the RTEC_2DIS model, the error correction could be executed in two steps: at-site correction and regional correction. At the first step, the error correction could be made by adding the gauged water-level forecasts into the corresponding forecast error estimated via the time-series approach and Kalman filtering algorithm with the bias of the estimations calculated in comparison to the observation as:

9 $${\varepsilon }_{corr}^{{t}^{*}}={h}_{obs}^{{t}^{*}}-{h}_{est}^{{t}^{*}}$$

10 $${\varepsilon }_{corr}^{{t}^{*}+1}={\varepsilon }_{TS}^{{t}^{*}+1}+{\varepsilon }_{KF}^{{t}^{*}+1}$$

where ${h}_{obs}^{{t}^{*}}and {h}_{est}^{{t}^{*}}$ represent the observed and estimated water level at the previous time step ${t}^{*}$ with a bias ${\varepsilon }_{corr}^{{t}^{*}}$, respectively; and ${\varepsilon }_{corr}^{{t}^{*}+1}$ ${\varepsilon }_{TS}^{{t}^{*}+1} and {\varepsilon }_{KF}^{{t}^{*}+1}$ account for the average error forecasts at the lead time $({t}^{*}+1$) with the forecast errors ${\varepsilon }_{TS}^{{t}^{*}+1} and {\varepsilon }_{KF}^{{t}^{*}+1}$ estimated by the time series and Kalman filtering methods, respectively; the resulting average from the auto-regression and moving-average of the fires three orders are employed in the estimation of the forecast error regarding the water levels at the gauged locations^49^. Figure 4(1) presents the process of correcting the water-stage estimates via the RTEC_TS&KF method with the used equations for calculating the forecast error through the time series and Kalmen filtering approaches.

Regarding the ungauged water-level estimates, the regional error correction could be conducted by estimating the corresponding forecast error to the ungauged water-level estimates via the Kriging equation with the weighted semivariogram functions^50^; the corrected water-level estimates at the ungauged locations could be obtained by adding the estimation into the above forecast errors (see Fig. 4(2). From Fig. 5(2), the forecast errors of the water-level estimates could be achieved via the RTEC-2DIS model.

Overall, when proceeding with 2D inundation simulations via the proposed SM_EID_2D model, the resulting inundation depths ${\widehat{h}}_{IOT}^{t+1}$ and ${\widehat{h}}_{EST,VIOTj}^{t}$ at the IOT-based and VIOT-based grids should be corrected via the RTEC_2DIS model with the difference in the observation and estimations at the previous time steps. In detail, the forecast error of the inundation depths at the IOT-based grids (${\varepsilon }_{IOT,corr}^{{t}^{*}+1}$) is calculated in advance using Eq. (9) adopted in the RTEC_TS&KF model, and they would be used in the calculation of the inundation-depth forecast errors at the VIOT-based grids (${\varepsilon }_{VIOT,corr}^{{t}^{*}+1}$) through the Kriging equations; By combining the inundation-depth estimates at the IOT-based and VIOT-based grids into the corresponding forecast errors, the resulting corrected inundation-depth estimates could be achieved as follows:

11 $${\widehat{h}}_{IOT,corr}^{t+1}={\widehat{h}}_{IOT}^{t+1}+{\varepsilon }_{IOT,corr}^{{t}^{*}+1}$$

12 $${\widehat{h}}_{VIOTj,corr}^{t+1}={\widehat{h}}_{VIOTj}^{t+1}+{\varepsilon }_{IOT,corr}^{{t}^{*}+1}$$

in which ${\widehat{h}}_{IOT,corr}^{t+1}$ and ${\widehat{h}}_{VIOTj,corr}^{t+1}$ account for the corrected inundation-depth estimates at the IOT-based and VIOT-based grids with the forecast errors, ${\varepsilon }_{IOT,corr}^{{t}^{*}+1}$ and ${\varepsilon }_{IOT,corr}^{{t}^{*}+1}$, respectively , quantified by means of the RTEC_2DIS model.

### Quantification of model performance

As mentioned earlier, the proposed SM_EID_2D model is trained using a considerable number of rainfall-induced inundation simulations reproduced via the SOBEK model with a significant number of simulated gridded rainstorms. To demonstrate the performance of the proposed SM_EID_2D model in the 2D inundation simulation, the model performance could be made in comparison to results from the SOBEK model (called the validated data), including the difference in the gridded inundation depths and flooding extent in time and space. Also, the root-mean-square-error (RMSE) is a commonly used performance index to quantify the difference between the estimations and observations; thus, the accuracy of the resulting gridded inundation depths from the proposed SM_EID_2D model in comparison to the validated data provided by SOBEK model can be quantified as:

13 $${RMSE}_{ID}=\sqrt{\frac{{\sum }_{t=1}^{{N}_{dur}}{\left({\widehat{h}}_{SM\_EID\_2D}^{t}-{\widehat{h}}_{SOBEK}^{t}\right)}^{2}}{{N}_{dur}}}$$

14 $${RMSE}_{FA}=\sqrt{\frac{{\sum }_{t=1}^{{N}_{dur}}{\left({\widehat{A}}_{SM\_EID\_2D}^{t}-{\widehat{A}}_{SOBEK}^{t}\right)}^{2}}{{N}_{dur}}}$$

where ${RMSE}_{ID}$ and ${RMSE}_{FA}$ denote the root mean square error of the estimated inundation depths and flooding time for the specific simulation cases of a given duration (*N*_*dur*_*)* through the proposed SM_EID_2D and SOBEK models, respectively.

To evaluate the temporal varying trend in the gridded inundation depths and flooding area, the correlation coefficients could be calculated as:

15 $${\rho }_{ID}=\frac{{\sum }_{t=1}^{{N}_{dur}}{({\widehat{h}}_{SM\_EID\_2D}^{t}-{\overline{\widehat{h}} }_{SM\_EID\_2D})\left({\widehat{h}}_{SOBEK}^{t}-{\overline{\widehat{h}} }_{SOBEK}\right)}}{{s}_{{\widehat{h}}_{SM\_EID\_2D}}{s}_{{\widehat{h}}_{SOBEK}}}$$

16 $${\rho }_{FA}=\frac{{\sum }_{t=1}^{{N}_{dur}}{({\widehat{A}}_{SM\_EID\_2D}^{t}-{\overline{\widehat{A}} }_{SM\_EID\_2D})\left({\widehat{A}}_{SOBEK}^{t}-{\overline{\widehat{A}} }_{SOBEK}\right)}}{{s}_{{\widehat{A}}_{SM\_EID\_2D}}{s}_{{\widehat{A}}_{SOBEK}}}$$

in which ${\rho }_{ID}$ and ${\rho }_{FA}$ stand for the correlation coefficients of the estimated inundation depth and flooding extent; and ${\widehat{h}}_{SM\_EID\_2D}^{t}$ and ${\widehat{A}}_{SM\_EID\_2D}^{t}$ denote the resulting gridded inundation depths and flooding area with the mean values (${\overline{\widehat{h}} }_{SM\_EID\_2D}$ and ${\overline{\widehat{A}} }_{SM\_EID\_2D}$) and standard deviations (${s}_{{\widehat{h}}_{SM\_EID\_2D}}$ and ${s}_{{\widehat{A}}_{SM\_EID\_2D}}$) from the proposed SM_EID_2D model; and ${\widehat{h}}_{SOBEK}^{t}$ and ${\widehat{A}}_{SOBEK}^{t}$ denote the estimations of the gridded inundation depths and flooding area with the mean values (${\overline{\widehat{h}} }_{SOBEK}$ and ${\overline{\widehat{A}} }_{SOBEK}$) and standard deviations (${s}_{{\widehat{h}}_{SOBEK}}$ and ${s}_{{\widehat{A}}_{SOBEK}}$) by the SOBEK model as the validated data.

As well as quantifying the difference in the inundation depth and flooding area, the performance indices are used for assessing the spatial change in the 2D inundation region by comparing the difference in the number of the grids with the nonzero and zero simulated inundation depths, defined as the inundated and non-inundated grids, respectively^4^ as:

17 $${{\theta }_{1}=\mathrm{Precision}}_{\mathrm{inundated}}= \frac{{N}_{IG\_JOINT}}{{N}_{IG\_SM\_EID\_2D}}$$

18 $${\theta }_{2}=\mathrm{Recall}=\frac{{N}_{IG\_JOINT}}{{N}_{IG\_SOBEK}}$$

19 $${\theta }_{3}={Precision}_{Available}=\frac{{N}_{IG\_JOINT}+{N}_{NIG\_JOINT}}{{N}_{VIOT}}$$

where *N*_*IG_SM_EID_2D*_ and* N*_*IG_SOBKE*_ serve as the number of the inundated grids detected via the proposed SM_EID_2D and SOBEK model, respectively; ${N}_{IG\_JOINT}$ and ${N}_{NIG\_JOINT}$ stand for the number of inundated grids and non-inundated identified both by the SM_EID_2D and SOBEK models; and NVIOT is the number of the available grids, including the IOT-based and VIOT-based grids. Of the four performance indices, the precision index for simulating inundated grids ${\theta }_{1}$ account for the accuracy of the inundated grids recognized by the proposed SM_EID_2D model: a high index ${\theta }_{1}$ indicates that the proposed SM_EID_2D model can provides the practical inundated grids with high accuracy. The recall index ${\theta }_{2}$ implies that the reliability of the resulting inundated grids from the proposed SM_EID_2D model can also be the inundated ones by the SOBEK model; a high index ${\theta }_{2}$ reveals that the proposed SM_EID_2D model can capture the practical inundated grids with high likelihood. In addition to the performance indices for evaluating the accuracy and reliability of the estimated inundated grids, the precision index for simulating available grids ${\theta }_{3}$ signifies that the accuracy of the spatial distribution of the resulting inundated and non-inundated grids from the proposed SM_EID_2D model, which could be combined as a flooding map; a high index ${\theta }_{3}$ indicates that the proposed SM_EID_2D model could emulates the spatial distribution of the available grids with an excellent fitness to the results from the SOBEK model.

### Model framework

Altogether, the development and application of the proposed SM_EID_2D model can be grouped into five parts: (1) Configuration of the hydrodynamic model for the 2D inundation simulation; (2) simulation of the gridded rainstorms; (3) Execution of the 2D rainfall-induced inundation simulation; (3) Establishment of the at-site ANN-based relationship between the rainfall and induced inundation depths; (4) Derivation of the regional ANN-based relationship between the inundation depths at the IOT-based and VIOT-based grids; (5) Copulation with the real-time error correction model for the 2D inundation simulation. Note that Steps 1–4 focus on the model development, while the remaining Step 4 proceeds with the model application. The detained framework of the model development and application are introduced as follows:

#### Model development

- *Step 1*: Collect the gridded hyetographs of historical rainstorm events and corresponding inundation-depth measurements at the IoT sensors in the study area.
- *Step 2*: Extract their gridded characteristics from the historical rainstorms, i.e., rainfall duration, gridded rainfall depth, an areal average of the cumulative dimensionless rainfall, and the associated bias and quantify their uncertainties in time and space in terms of the statistical properties.
- *Step 3*: Generate a considerable number of rainfall fields with high spatiotemporal resolutions comprised of the simulated gridded rainfall characteristics by the SM_GSTR model with the statistical properties of gridded rainfall characteristics extracted at Step 2.
- *Step 4*: Carry out the 2D inundation simulation via the SOBEK model with a significant number of gridded rainstorms simulated at Step 3 to achieve the simulated inundation depths at the IoT sensors (IOT-based grids) and ungauged locations (i.e., VIOT-based grids);
- *Step 5*: Extract the simulations of the gridded rainfalls and the inundation depths at the specific time steps at the IOT-based and VIOT-based grids and calculate their areal average rainfall using the simulated rainstorms at the grids under the conditions of spatial and temporal resolutions, 3 km and 3 h.
- *Step 6*: Train the ANN-GA-SA_MTF model to establish the relationships (Eq. (6)) between the areal average rainfall and induced inundation depths at the IOT-based grids through Eq. (5).
- *Step 7*: Train the ANN-GA-SA_MTF model to derive the relationships (i.e., Eq. (8)) between the inundation depths at the VIOT-based grids and spatial average of the inundation depths calculated with estimated inundation ones at the IOT-based grids using Eq. (7).

#### Model application

After training the ANN_GA-SA_MTF model for the relationship in estimating the inundation depths at the IOT-based and VIOT-based grids via Eqs. (6) and (8), the inundation depths of 1-h lead time could be accordingly achieved under consideration of the rainfall observations and forecasts as well as the observed inundation depths at the IoT sensors for the forward three hours; the above estimation procedure could be referred to the following framework:

- *Step 1*: Collect the gridded rainstorm observation of the forward three hours and forecasts of 1-h lead time as well as the observed inundation depths of the forward three time steps at the IOT-based grids during the rainfall-induced flood events
- *Step 2*: Estimate the inundation depths of the 1-h lead time at the IOT-based grids within the study area from Eq. (6) configured in the proposed SM_EID_2D model.
- *Step 3*: Compute the corresponding spatial averages of the inundation-depth estimates at the IOT-based grids to the target VIOT-based grids via the inverse-distance method (Eq. (7)).
- *Step 4*: Estimate the inundation depths at the VIOT-based grids via Eq. (8) adapted in the proposed SM_EID_2D model with the spatial average of the inundation depths obtained at Step 3.
- *Step 5*: Perform the real-time correction for the resulting inundation-depth estimates at the IOT-based and VIOT-based grids with the RTEC_2DIS method coupled in the proposed SM_EID_2D model based on the inundation-depth estimates in comparison to the real-time measurements at the IoT sensors obtained at Step 4.
- *Step 6*: Delineate the potential inundation region with the corrected inundation-depth estimates at all available grids (IOT-based and VIOT-based grids) and summarize the number of inundated grids to quantify the corresponding flooding extent

## Study area and data

Miaoli County is located in western Taiwan with borders of the Taiwan Strait to the west (see Fig. 5) with two rivers (Houlong River and Zhonggang River). Houlong River is the biggest river with a watershed area (nearly 537 km^2^), and its length approximates 58.3 km. Also, in the Miaoli County, there are eighteen townships in which Miaoli City is the capital of the county, selected as the study area in this study.

Various hydrological measurement gates within Miaoli County have been set up, including 65 rain-gauges, three water-level stations, and two reservoirs (Min-Te and Liyu-Lake). Moreover, 22 roadside IoT sensors are set up to real-time detect the inundation depths, symbolled as the red points, with three IoT sensors within the study area (Miaoli City) (defined IOT-based grids). In addition to the hydrological measurement gates, as shown in Fig. 5, 1045 radar-rainfall-related grids are configured by the Taiwan Central Weather Bureau (CWB) to supply the rainfall data of high temporal and spatial resolutions of 15 min and 1.5 km, respectively; among the radar-rainfall related grids, 22 grids are located in the Miaoli City marked with the blue circles.

**Figure 6:**
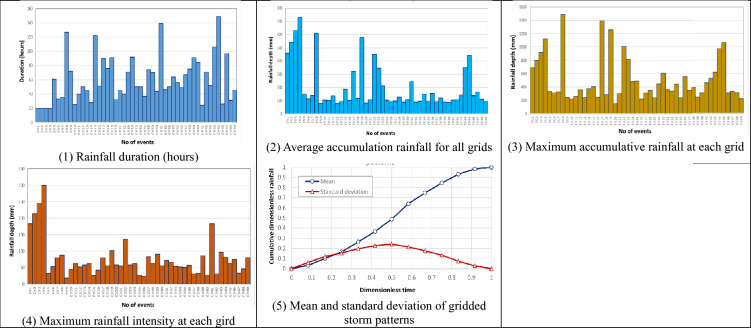
Summary of the gridded rainfall characteristics from 50 historical rainstorms in Miaoli County^4^.

**Figure 7:**
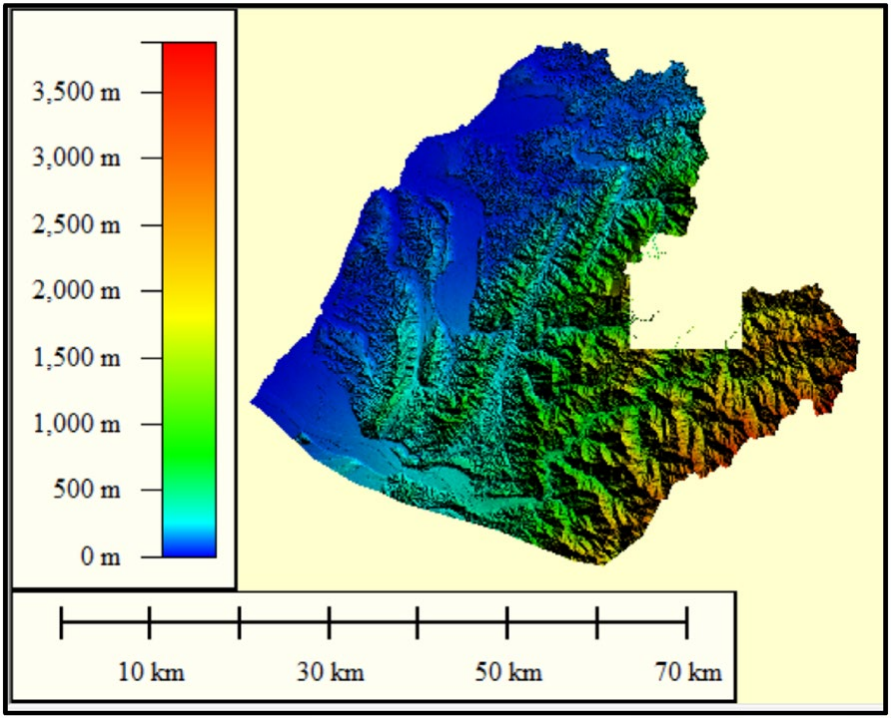
Digital elevation map (DEM) of Miaoli County^4^; this figure is created using Global Mapper, URL: https://www.bluemarblegeo.com.

**Figure 8:**
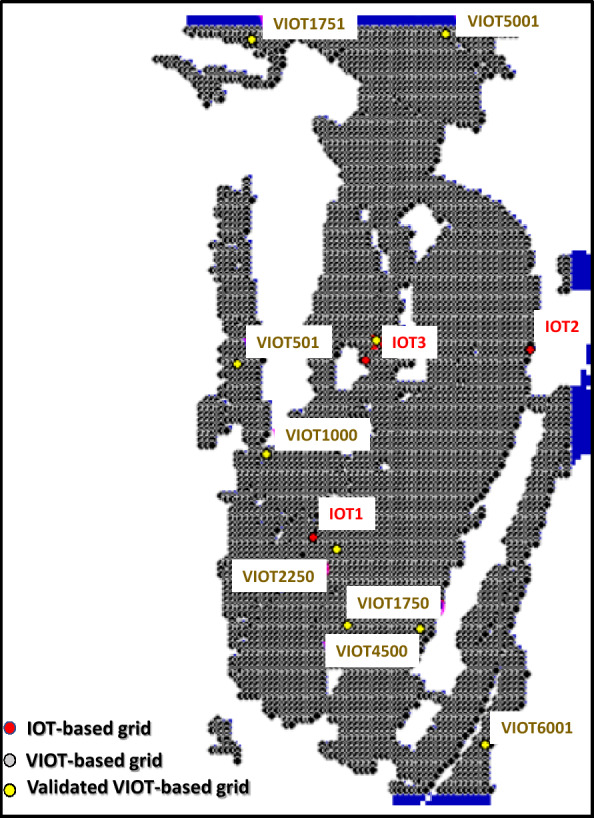
Locations of IOT-based and VIOT-based grids within Miaoli City.

To develop the proposed SM_EID_2D model, 50 gridded rainstorm events recorded from 2009 to 2018 are used in the simulations of a significant number of rainfall-induced flood events, treated as the training and validating datasets. Figure 6 presents the gridded rainfall characteristics extracted from 50 historical rainstorms. Furthermore, in simulating the rainfall-induced flood events, the digital elevation map (DEM) should be given to describe the flow paths caused by the rainstorms; in this study, the DEM of Miaoli County (see Fig. 7) is utilized in the 2D rainfall-induced inundation simulations as the training dataset. Figure 8 shows that the west of Miaoli County is a plain region with the other side of the alpine zone. This indicates that the spatial high variation significantly exists in the elevation within the study area, Miaoli County. Specifically, according to the digital elevation map (DEM) of 40 m for the study area Miaoli City, it can be seen that 6823 available girds, which are inundated with high likelihood, are located in Miaoli City, which are named the VIOT-based grids (see Fig. 8).

**Figure 9:**
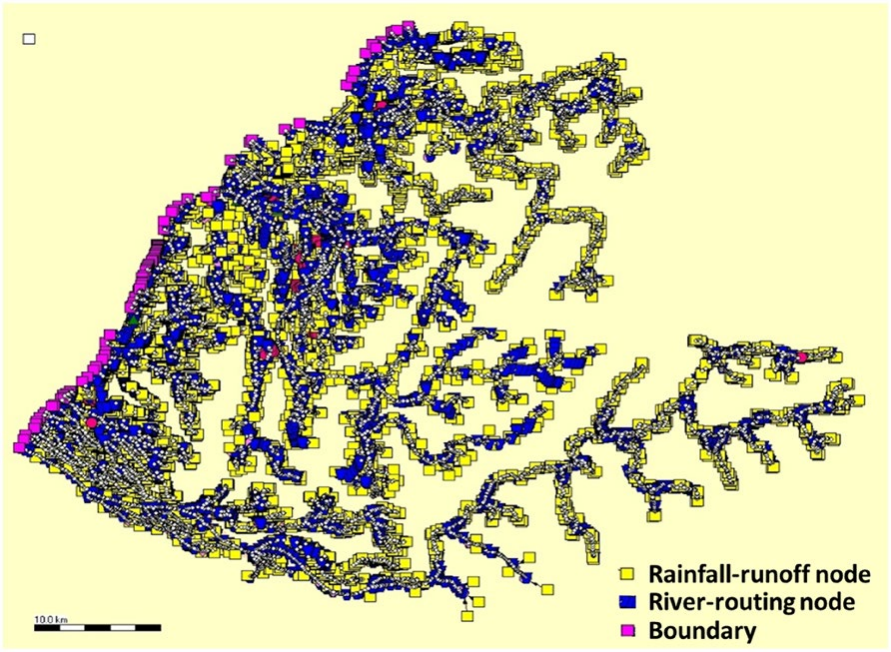
2D SOBEK model for Miaoli County (note: Circle is the rainfall-runoff computing node for each sub-basin)^4^.

In this study, using the above 40 m $\times 40$ m DEM in Miaoli County (see Fig. 7), the SOBEK 1D-2D hydrodynamic model could be configured with a variety of hydraulic structures, including the drainage channel, the pumping stations, sewer system, and draining gates; thus, the resulting SOBEK model for the Miaoli County could be applied in the 2D inundation simulation as shown Fig. 9, indicating that the computation objects for emulating the hydrological and hydraulic analysis are summarized in Table 2 in which the rainfall-runoff models (e.g., SCS-UH and SAC-SMA) are adapted in the SOBEK mode to estimate the rainfall-induced runoffs as the boundary conditions of the hydrodynamic routing. Within the SOBEK model, the calibrated parameters could be grouped into two types: hydraulic and hydrological parameters. The hydraulic parameters (i.e., the roughness coefficients in the riverbed and urban) could be determined based on the topographic data and land use. As for the parameters of the rainfall-runoff (RR) model, the historical data, including the rainfall and induced runoff, are required to calibrate the RR parameters via the optimization method (e.g., Genetic algorithm), which could be referred to the investigation by Wu et al.^43^.

**Table 2:** Computation objects adapted in the SOBEK model for Miaoli County^4^.

Function	Facilities	Number
Hydraulic analysis	Sub-basins	4731
	Cross-sections	9838
	Gates	62
	Bridges	9018
	Sewer	68.6 km
	Manholes for sewer system	1382
Hydrological analysis	Rainfall-runoff node	4097

## Results and discussion

This study aims to develop an ANN-derived model for carrying out 2D inundation simulation with the rainfall observation and forecasts and the observed inundation depth at the IoT sensors to estimate the ungauged inundation depths, called the SM_EID_2D model. In the proposed SM_EID_2D model, two types of relationships between the gauged inundation depth with the areal average rainfall and ungauged inundation depths, respectively, which are configured in terms of the ANN-derived, ANN_GA-SA_MTF model^36^ (i.e., Eqs. (6) and (8)); the above ANN-GA-SA_MTF is configured with three neurons in a hidden layer for the three IOT-based grids and 6823 VIOT-based grids; it could be derived and demonstrated using a significant training dataset. The resulting training dataset is from a considerable number of rainfall-induced inundation simulations achieved via the SOBEK model with the generated gridded rainstorms. The relevant results from the model development and validation are addressed below:

### Simulation of rainfall-induced flood events

To train the aforementioned two ANN-GA-SA_MTF models adapted in the proposed SM_EID_2D model, 1000 simulations of rainfall-induced flood events should be achieved as the training datasets in advance. According to Section "Simulations of training datasets, including rainfall-induced inundations", 1000 generations of the gridded rainstorm events are supposed to be made via the SM_GSTR model with the gridded rainfall characteristics extracted from 50 historical events as shown in Fig. 6^4^; the 2D inundation simulation could then be carried out via the SOBEK for the study area to obtain 1000 simulation cases of the inundation- depth hydrographs at the IOT-based and VIOT-based grids. The detailed process of producing the training datasets is expressed as follows:

While generating the gridded rainstorms via the SM_GSTR model, the gridded rainfall characteristics are supposed to be extracted from the 50 historical events, as shown in Fig. 7, indicating that the average of the event-based duration reaches nearly 60 h with the minimum and maximum of 20 h and 150 h, respectively. Furthermore, the gridded rainfall depths' spatial averages are approximately 100 mm and 800 mm. Apart from the rainfall duration and depth, the rainfall intensity, on average, approximates 80 mm/hr with a high coefficient of variance (CV) of 0.75; also, the maximum of the spatial average of the dimensionless rainfall regarding the gridded storm pattern) reaches 0.16 with a standard deviation of 0.12 (i.e., CV = 0.73), revealing that the storm patterns at various grids will likely exhibit a significant dispersion in space and time. In this study, uncertainty analysis is employed for the gridded rainfall characteristics to quantify their statistical properties, which are applied to reproduce 1000 simulations of gridded rainstorm events via the SM_GSTR model^37^.

**Figure 10:**
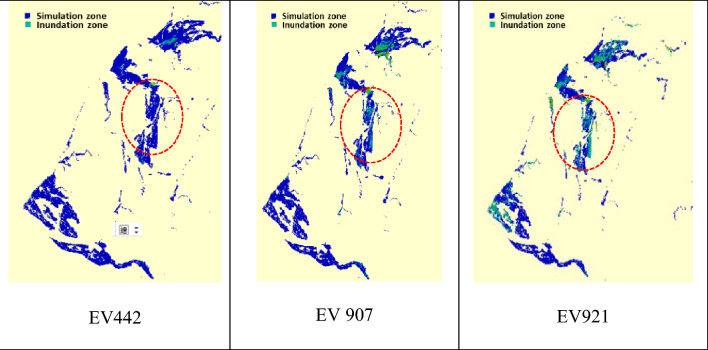
The maximum potential flooding zones for three simulated rainstorms in Miaoli County with the study area Miaoli City symbolled as a red circle^4^.

Using the resulting 1000 simulations of the gridded rainstorms, the corresponding rainfall-induced 2D inundation scenarios could be simulated via the SOBEK model derived based on the hydrological and topographic data in the Miaoli County, in which the detail configuration could be referred to in Section "Study area and data". Eventually, 1000 simulations of the gridded inundation depths and corresponding flooding extents could be achieved Fig. 10. The maximum potential flooding zones for three simulated rainstorms in Miaoli County with the study area Miaoli City symbolled as a red circle^4^.

### Model configuration

According to the model framework introduced in Section "Methodology", the proposed SM_EID_2D model could be developed by configuring the ANN-GA-SA_MTF model with a hidden layer at the IOT-based and VIOT-Based grid with the 7 and 1 input factors, respectively, via Eqs. (6)–(8); the definitions of the remaining parameters of the ANN_GA-SA_MTF model could be referred to in Table 3. Additionally, since the study area Miaoli City includes three roadside IoT sensors and 6823 ungauged grids, three sets and 6823 sets of the parameters of the ANN_GA-SA_MTF models for the IOT-based and VIOT-based grids could be calibrated with the training datasets comprised of the 1000 simulations of the rainfall-induced flood events.

**Table 3:** Definition of the parameters of the ANN_GA-SA_MTF model used in the model development.

Parameters	Definition		
Transfer functions used	TF1-TF10		
Input factors	IOT-based grids	Average rainfall	${\overline{R} }_{IOT}^{t+1},{\overline{R} }_{IOT}^{t},{\overline{R} }_{IOT}^{t-1},{\overline{R} }_{IOT}^{t-2}$
		Inundation depth	${h}_{IOT}^{t},{h}_{IOT}^{t-1},{h}_{IOT}^{t-2}$
	VIOT-based grids	Inundation depth	${\overline{h} }_{IDW,VIOT}^{t}$
Output factor	IOT-based grids	Inundation depth	${\widehat{h}}_{IOT}^{t+1}$
	VIOT-based grids		${\widehat{h}}_{EST,VIOT}^{t}$
Number of hidden levels	1		
Number of neurons	IOT-based grids	8	
	VIOT-based grids	3	
Calibration of parameters of transfer function	Number of optimizations	10	
	Weights of neurons (${\upomega }_{HL})$	Mean	1
		Standard deviation	3
	Bias of function (${\uptheta }_{TF})$	Mean	0
		Standard deviation	1
	Adjusting factor (${\propto }_{TF})$	Mean	1
		Standard deviation	0.005

#### IOT-based grids

While training the ANN_GA-SA_MTF models for the IOT-based grids, the areal average rainfall and corresponding simulated inundation depths at the IOT-based grids are supposed to be selected from the training datasets. By referring to the results of the investigation from Wu et al.^13^, in this study, the subbasins involving the IOT-based grids are recognized based on the locations of the IoT sensors; their corresponding areal average rainfall via Eq. (5) at the specific time steps during the simulated events, equal to the ratio of 0.3, 0.4, 0.5, 0.7, 0.8 and 0.9 multiplied by the durations, are treated as the training datasets. Table 4 lists the calibrated parameters of the ANN_GA-SA_MTF models for the IOT-based grids with significant spatial dispersion.

**Table 4:** The calibrated parameters of the ANN_GA-SA_MTF models for the IOT-based grids.

IOT-based grid	Adjust factor $({\propto }_{{\varvec{T}}{\varvec{F}}})$	1.00725									
IOT1 TWD97_X: 232275.81 TWD97_Y:2717317.41	Weights of neurons ${{\varvec{\upomega}}}_{{\varvec{H}}{\varvec{L}}}$	The 1st hidden layer		Input factors							
				1	2	3	4	5	6	7	Bias
		Neuron	1	4.698	0.463	3.349	2.880	1.134	− 1.644	− 0.279	− 5.502
			2	4.414	3.041	3.446	4.039	6.038	− 2.478	4.420	− 0.323
			3	− 0.413	3.321	− 1.787	0.149	− 5.275	6.957	4.963	2.838
			4	− 0.357	1.730	1.380	− 1.427	− 6.280	2.645	− 2.797	− 3.958
			5	3.051	− 4.753	− 0.050	1.843	− 3.907	1.823	4.547	− 1.920
			6	6.353	2.829	− 1.773	0.447	2.969	0.515	− 2.619	0.595
			7	3.313	6.194	2.162	3.379	4.064	− 0.442	− 0.759	1.036
			8	− 2.309	0.069	0.481	1.613	8.440	0.987	1.111	1.670
		Output layer		The 1st hidden layer							
				1	2	3	4	5	6	7	Bias
		Input factor	1	2.225	− 1.462	1.238	0.968	− 0.273	0.804	− 0.894	− 0.170

IOT-based grid	Adjust factor $({\propto }_{{\varvec{T}}{\varvec{F}}})$	0.99823									
IOT2 TWD97_X: 234020.45 TWD97_Y: 2718817.47	Weights of neurons ${{\varvec{\upomega}}}_{{\varvec{H}}{\varvec{L}}}$	The 1st hidden layer		Input factors							
				1	2	3	4	5	6	7	Bias
		Neuron	1	0.490	0.076	8.571	7.611	3.639	4.709	1.623	0.780
			2	− 0.139	1.860	0.476	0.283	2.932	− 1.594	5.459	0.013
			3	− 1.672	2.273	− 1.901	6.580	− 2.573	0.513	1.588	0.126
			4	− 0.276	− 0.414	2.088	8.163	1.232	1.606	1.249	2.080
			5	2.974	0.066	− 3.116	1.806	1.603	0.443	3.002	− 0.276
			6	− 3.201	1.952	2.449	− 2.156	0.779	− 0.975	− 0.691	3.637
			7	4.149	1.551	0.578	2.694	1.458	1.294	3.421	1.246
			8	− 9.457	1.545	− 0.345	4.410	0.849	− 5.729	0.324	− 0.565
		Output layer		The 1st hidden layer							
				1	2	3	4	5	6	7	Bias
		Input factor	1	− 1.242	− 0.161	− 1.279	− 1.295	− 0.931	0.702	− 1.767	− 0.112

IOT-based grid	Adjust factor $({\propto }_{{\varvec{T}}{\varvec{F}}})$	1.00725									
IOT3 TWD97_X: 232687.46 TWD97_Y: 2718740.68	Weights of neurons ${{\varvec{\upomega}}}_{{\varvec{H}}{\varvec{L}}}$	The 1st hidden layer		Input factors							
				1	2	3	4	5	6	7	Bias
		Neuron	1	0.741	1.407	− 1.350	− 0.451	0.388	0.545	2.041	− 1.227
			2	3.023	2.918	0.709	3.363	− 0.374	2.736	0.061	− 3.650
			3	1.843	4.658	2.836	− 1.465	0.282	2.387	− 0.482	4.742
			4	− 2.906	8.361	− 0.918	2.958	0.472	3.267	1.681	− 1.842
			5	0.667	1.054	− 4.314	− 1.562	− 0.725	− 1.232	1.460	− 1.744
			6	6.944	− 0.823	− 2.039	0.126	4.333	− 0.028	− 1.348	− 0.953
			7	− 0.333	1.585	3.867	− 2.577	1.211	− 2.078	− 1.843	− 1.882
			8	0.900	− 1.559	− 2.952	3.739	1.931	1.284	− 0.135	2.730
		Output layer		The 1st hidden layer							
				1	2	3	4	5	6	7	Bias
		Input factor	1	− 0.389	0.895	− 1.246	− 1.736	0.234	0.130	0.531	− 0.563

#### VIOT-based grids

Thereby, similar to the IOT-based grids, using the parameter definition shown in Table 4, the ANN_GA-SA_MTF model for estimating the inundation depths at the VIOT-based grids can be developed by training the ANN_GA-SA_MTF model with 1000 simulations of the inundation depths at the aforementioned specific time steps at all grids. Table 5 illustrates the calibrated parameters of the ANN_GA-SA_MTF model for the 501st, 1000th, and 5001st VIOT-based grids. Thus, the resulting model parameters from the model training for the ANN_GA-SA_MTF models at the VIOT-based grids with a significant spatial variation could be observed, implying that the SM_EID_2D model could reasonably reflect and describe the varying trend of the rainfall0indued inundation depth with the locations of the grids of interest.

**Table 5:** The calibrated parameters of the ANN_GA-SA_MTF models for the VIOT-based grids.

VIOT-based grid	Adjust factor $({\propto }_{{\varvec{T}}{\varvec{F}}})$	0.99708					
501st grid TWD97_X: 231658.5 TWD97_Y: 2718701	Weights of neurons ${{\varvec{\upomega}}}_{{\varvec{H}}{\varvec{L}}}$	The 1st hidden layer		Input factors			
				1	Bias		
		Neuron	1	5.39432	− 1.72881		
			2	1.64042	− 0.42381		
			3	5.90881	− 6.43761		
		Output layer		The 1st hidden layer			
				1	2	3	Bias
		Input factor	1	− 0.75616	− 0.49120	0.81154	− 0.23816

VIOT− based grid	Adjust factor $({\propto }_{{\varvec{T}}{\varvec{F}}})$	1.00623					
1000th grid TWD97_X: 231898.5 TWD97_Y: 2718021.0	Weights of neurons ${{\varvec{\upomega}}}_{{\varvec{H}}{\varvec{L}}}$	The 1st hidden layer		Input factors			
				1	Bias		
		Neuron	1	0.55883	− 1.34752		
			2	0.27115	− 2.15728		
			3	1.31376	− 3.41824		
		Output layer		The 1st hidden layer			
				1	2	3	Bias
		Input factor	1	0.99939	− 0.33276	1.20717	0.12047

VIOT-based grid	Adjust factor $({\propto }_{{\varvec{T}}{\varvec{F}}})$	0.99272					
5001th grid TWD97_X: 233338.5 TWD97_Y: 2721381.0	Weights of neurons ${{\varvec{\upomega}}}_{{\varvec{H}}{\varvec{L}}}$	The 1st hidden layer		Input factors			
				1	Bias		
		Neuron	1	1.88168	1.03727		
			2	3.11022	− 0.48199		
			3	6.36106	− 5.35567		
		Output layer		The 1st hidden layer			
				1	2	3	Bias
		Input factor	1	− 0.99511	− 0.81306	0.37511	− 0.79509

### Model validation

**Figure 11:**
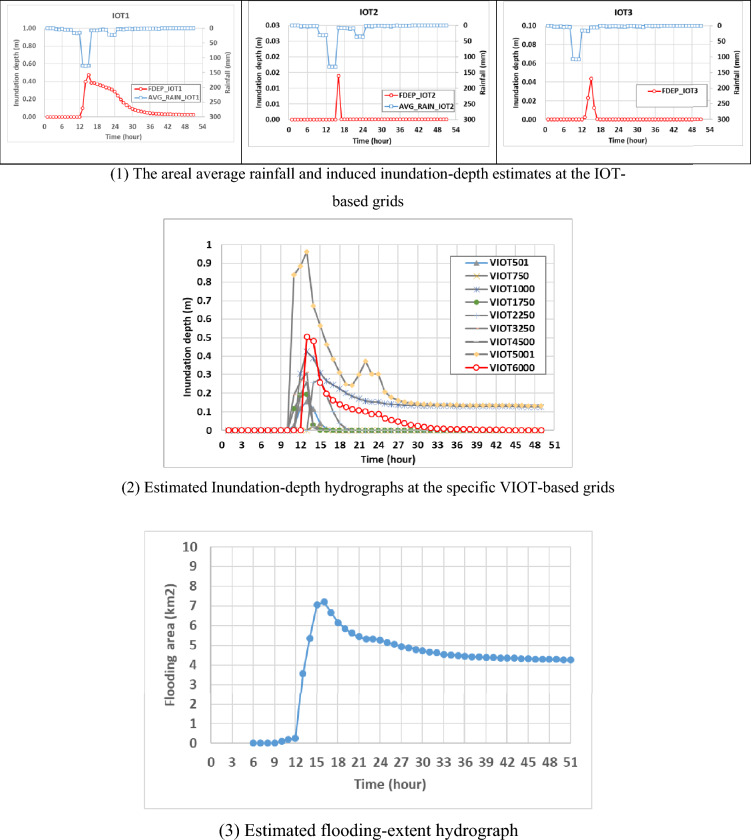
Hydrographs of the areal average rainfall, gridded inundation depths, and flooding extent selected from the validation event via the SOBEK model for the model demonstration.

In general, to quantify and assess the performance of the flood-related numerical models, their results would be compared to the observations. However, due to the lack of enough measurements related to the inundation in the study area (Miaoli City), the validation of the proposed SM_EID_2D model would proceed with comparing their estimations of the inundation depths at all grids and corresponding flooding extent to the results from the physically-based hydrodynamic numerical (SBOEK) model (named validated data). Note that the comparison should be done under identical rainfall conditions and topographical characteristics in time and space.; thus, the 921st simulated rainstorm event of 51 h, excluded from the training datasets, is selected as the validation event, which noticeably causes flood-induced inundation. Thus, Fig. 11 show the simulations of the gridded inundation depths and resulting flooding area and the extent for the validation event via the SBOEK model, which are treated as the validated data. In detail, Fig. 11(1) represents the simulated areal average rainfall and related inundation-depth hydrographs at the three IoT sensors (i.e., IOT-based grids) as well as the corresponding flooding area for the validation event, revealing that the rainfall mainly takes place between the 8th and 25th hours with the maximum rainfall intensities ranging from 20 mm/hour and 125 mm, attributed to the existence of spatial solid correlation; also, the corresponding inundation-depth estimates reach the maximum, 0.47 m (IOT1) 0.04 (IOT2) and 0.01 m (IOT3), at the 15th time step with a smooth decrease to 0.02 m (IOT1) and 0.0002 m (IOT3) at the end. Also, the estimated inundation depths at the specific 9 VIOT-based grids (see Fig. 11(2)) indicate their spatial varying trend assembles those at the IOT-based grids with the maximum ones at the around 15th hour, ranging from 0.15 to 0.95 m; also, apart from the 1000th and 5001st VIOT-basted grids, the inundation depth of which reach nearly 0.1 m at the end of validation event, the inundation depths at the reaming VIOT-based grids gradually drop to zero. Furthermore, given the resulting flooding extent with the gridded maximum inundation-depth estimates (see Fig. 11(3)), the flooding extent has a marked increase to the maximum (about 7 km^2^) at the nearly 15th hour. It then gradually drops to 4 km^2^ at the end.

In summary, the proposed SM_EID_2D model would be verified in terms of two parts: the difference in the gridded inundation depths as well as the corresponding flooding extents, and spatial distribution of inundation; the difference in the gridded inundation and flooding extent could be quantified by calculating the root mean square error (RMSE) and correlation coefficient using Eqs. (13)–(16); Furthermore, the evaluation of the spatial flooding distribution could be made by quantifying the model performance indices ${\theta }_{1}, {\theta }_{2} and {\theta }_{3}$ through Eqs. (17)–(19).

#### Gridded inundation depths

**Figure 12:**
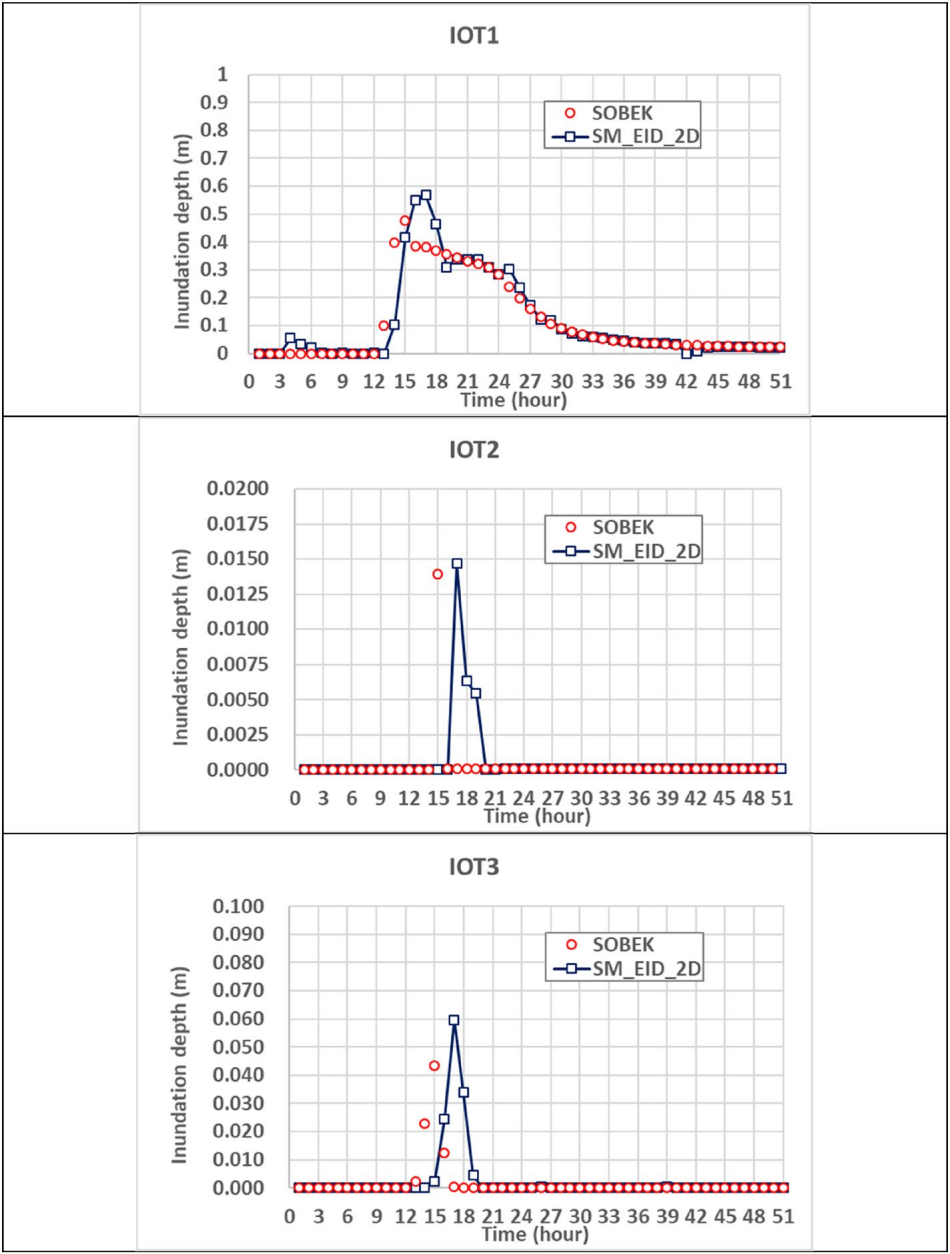
Comparison of the estimated inundation depths with the validated data at IoT-based grids.

While proceeding with the 2D inundation simulation via the proposed SM_EID_2D model configured for the study area, the inundation depths at three IOT-based grids should be estimated in advance via the ANN_GA-SA_MTF models with the corresponding parameters sets (see Table 4) with the corresponding areal average rainfalls (see Fig. 11(1)). Figure 12 shows the comparison of the estimated inundation depths with the validated data at IoT-based grids; their differences could be quantified using the root means square errors (RMSE) and correlation coefficients via Eqs. (13)–(16) as listed in Table 7.

As compared with the results from Fig. 5 and Table 6, the resulting RMSE values and correlation coefficients generally range between 0.001 m ~ 0.006 m and 0.5 ~ 0.92; on average, they reach 0.023 m and 0.75, respectively; this implies that the estimated inundation depths at the IOT-based grids have a good match with the validated data with a low RMSE and high correlation coefficient. In particular, at the IOT1 during the validation event with a significant temporal change in the water levels, the inundation depths between the 12th and 24th hours caused by higher hourly rainfalls obviously approach the validated ones with an excellent fitness to the temporal varying trend based on the low RMSE and high correlation coefficient, 0.06 m and 0.92, respectively.

**Table 6:** Statistical properties of the model performance indices for the estimated inundation depths and flooding area.

Inundation depth (m)		Root mean square error RMSE	Correlation coefficient
IOT-based grids	Mean	0.023	0.755
	Standard deviation	0.032	0.244
VIOT-based grids	Mean	0.101	0.623
	Standard deviation	0.066	0.118
Spatial average inundation depth		0.097	0.738
Maximum average inundation depth		3.249	0.493
Flooding extent (km^2^)		2.99	0.584

**Figure 13:**
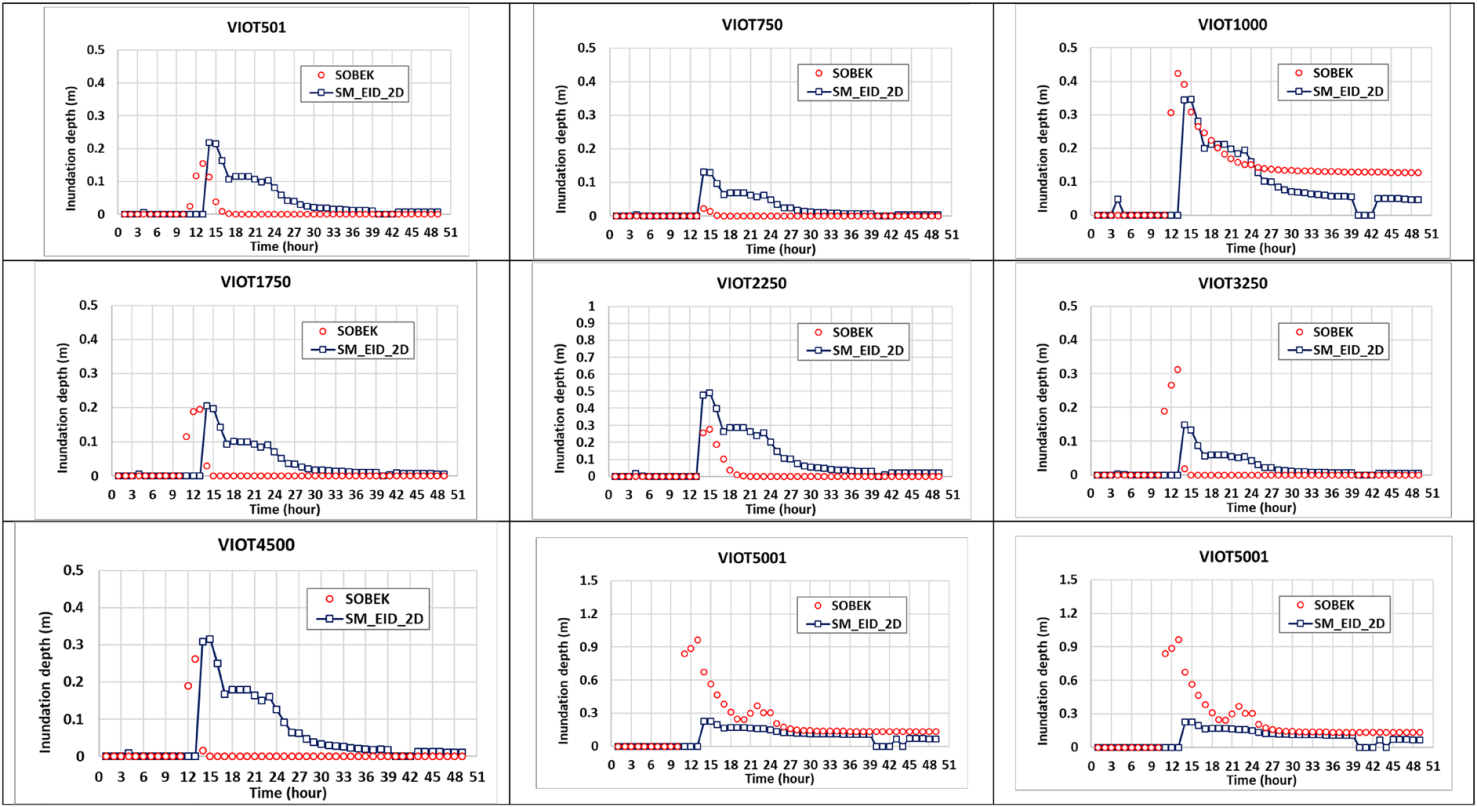
Comparison of the estimated inundation depths with the validated data at the VIOT-based grids of interest.

**Figure 14:**
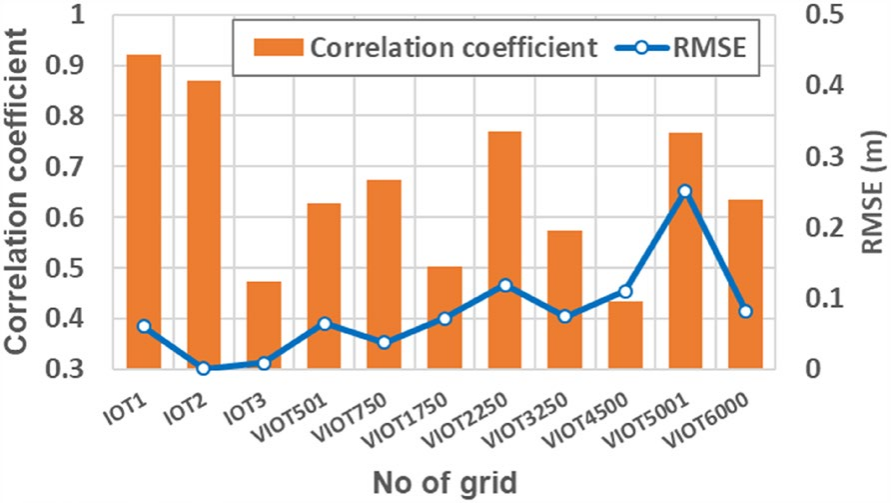
Performance indices of the estimated inundation depths at the IOT-based and VIOT-based grids.

As well as the comparison in the estimated inundation depths at three IOT-based grids, the model performance could be evaluated based on the results from the proposed SM_EID_2D model at 9 VIOT-based grids, the locations of which could be referred to in Fig. 8. Figures 12, 13 and 14 present the comparison of the estimated inundation depths via the proposed SM_EID_2D model with the calibrated parameters (see Tables 4 and 5) with the validated data at the VIOT-based grids of interest and the corresponding performance indices of the estimated inundation depths at the IOT-based and VIOT-based grids, respectively. According to Figs. 12, 13 and 14, the estimated inundation depths via the proposed SM_EID_2D model exhibit somewhat significant differences from the validated data, especially for the high rainfall intensity, with the RMSE ranging from 0.06 to 0.25 m (on average, 0.023 m), but they have similar temporal change with the validated data as a result of the correlation coefficients between 0.5 and 0.8 (on average 0.7); for example, regarding the 1000th and 5001st VIOT-based grids with the nonzero inundation depths at the end of the validation event, the estimated ones by the proposed SM_EID_2D model also reach a constant slightly less than the validated data. In particular, the proposed SM_EID_2D model inconsistently underestimates or overestimates the inundation depths at the VIOT-based grids of interest; for example, at the 750th VIOT-based grid, the estimated inundation depths via the proposed SM_EID_2D model are markedly greater than the validated data; in contrast, the underestimated inundation depths could be achieved at the 6000th VIOT-based grids, indicating that the proposed SM_EID_2D model could have the ability to provide the reliable inundation depths at the VIOT-based grids without the systematic bias.

**Figure 15:**
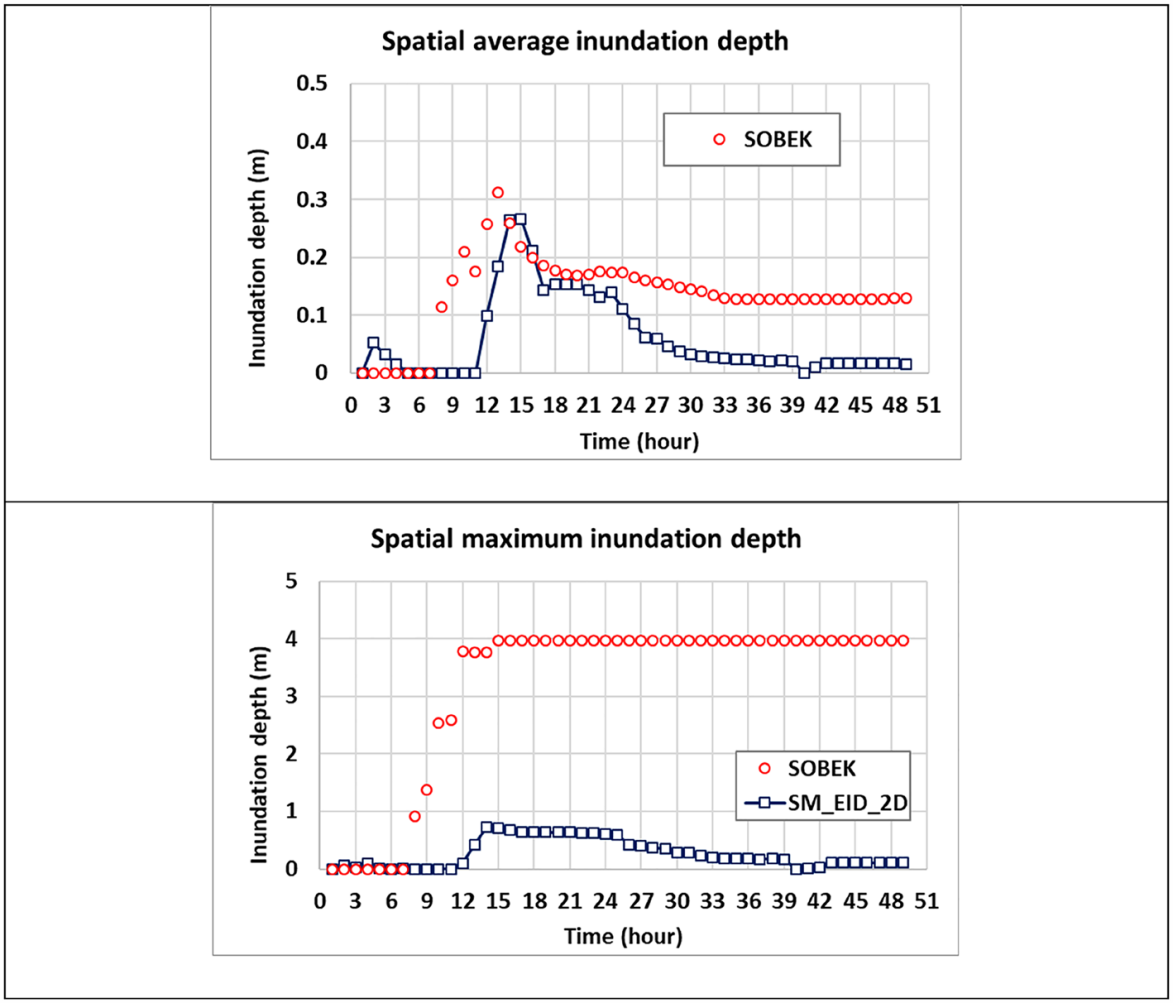
Comparison of the spatial average and maximum of the estimated inundation depths with the validated data.

Furthermore, to evaluate the spatial difference in the inundation depth, the spatial average and maximum of the estimated inundation depths at all available grids are calculated from the results from the proposed SM_EID_2D and SOBEK model, respectively, as shown in Fig. 15. Figure 15 and Table 6 present that the spatial average inundation depth matches the validated with a low RMSE of 0.097 m and a high correlation coefficient (i.e., 0.738). In total, the gridded inundation depths estimated by the proposed SM_EID_2D model approach the change in the validated data in space, sharply rising from 0 m to the maximum of 0.26 m (t = 15-h) and then slightly dropping to 0.02 m at the end of the event. However, as for the spatial maximum of the gridded inundation depth, the results from the proposed SM_EID model are considerably underestimated subject to the validated data with a significant RMSE of 3.24 mm, indicating that the maximum of inundation depths via the SOBEK model approximates 4 m; nonetheless, they exhibit a somewhat similar varying trend with a correlation coefficient of 0.5. This is because the SOBEK model is mainly configured in Miaoli County, covering the grids near the urban and fluvial zone, in contrast with the SM_EID_2D model only for Miaoli City; by so doing, the validated maximum inundation depths are more likely to be achieved in the river channel.

Overall, using the given gridded rainstorm, the proposed SM_EID_2D not only could provide more accurate estimated inundation depths at the IOT-based grids in response to the rainstorms with high likelihood but also could capture the temporal varying trend in the gridded inundation depths at the VIOT-based grids with an acceptable bias and without considering the systematic bias.

#### Flooding extent

**Figure 16:**
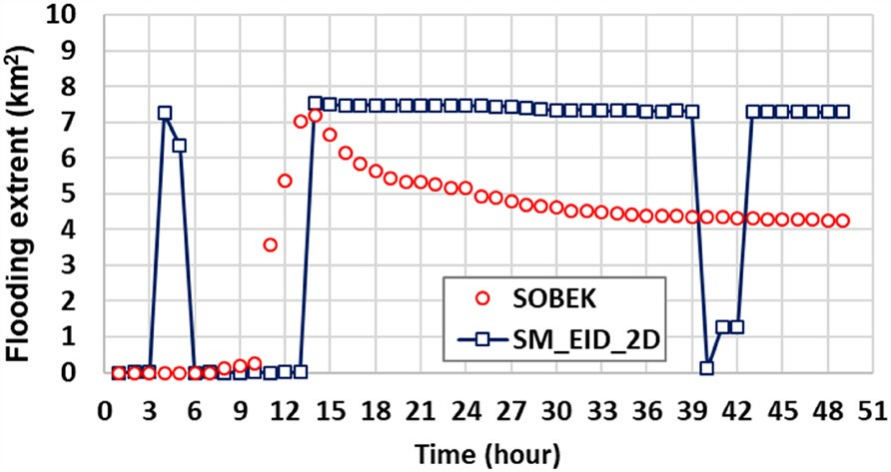
Comparison of the estimated flooding extent with the validated data.

As well as evaluating the difference in the gridded inundation depths, the resulting flood extent related to the grid size multiplied by the number of inundated grids identified via the proposed SM_EID_2D model could be evaluated as shown in Fig. 16. Note that in this study, the nonzero water levels are treated as the inundation depth; namely, the inundation-depth threshold is 0 m. Figure 16 shows the comparison of the estimated flooding extent via the proposed SM_EID_2D model with the validated data; it can be seen that the estimated flooding extents both by the SOBEK and proposed SM_EID_2D model area reach the maximum of 7 km^2^ nearly after the 31^st^ hour with a modest bias (0.3 km^2^); however, the resulting flooding extent from the proposed SM_EID_2D slightly decreases by 0.23 km^2^; instead, the validated data significantly drops to 4 km^2^. Also, although the flooding extents are significantly overestimated at the 4th and 5th hours, they are mainly caused by the modest inundation depths (nearly 0.02 m), lower than the validated data (around 0.15 m) as shown in Fig. 15. That is to say, the flooding area calculated by the proposed SM_EID_2D model slightly differs from the validated data provided by the SOBEK model with a RMSE of 2.99 km^2^; also, their correlation coefficient nearly reaches 0.6, implying that the proposed SM_EID_2D could reflect the temporal change pattern in the flooding extent triggered by heavy rainfall with an acceptable reliability.

Therefore, except for the estimated flooding area at the recession with a significant difference (nearly 3 km^2^), the resulting flooding extent from the proposed SM_EID_2D model has a good agreement with validated data estimated by the SOBEK model. As a result, the proposed SM_EDD_2D model exhibits good ability to capture the varying trend of the validation data in time under an acceptable bias, especially for the maximum flooding extent.

#### Spatial distribution of inundation simulation

**Figure 17:**
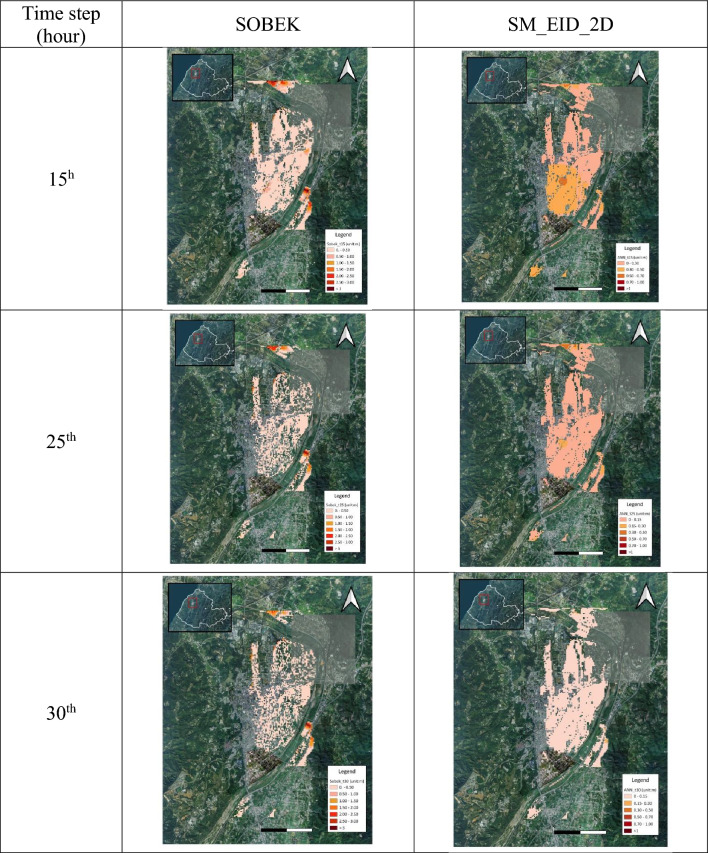

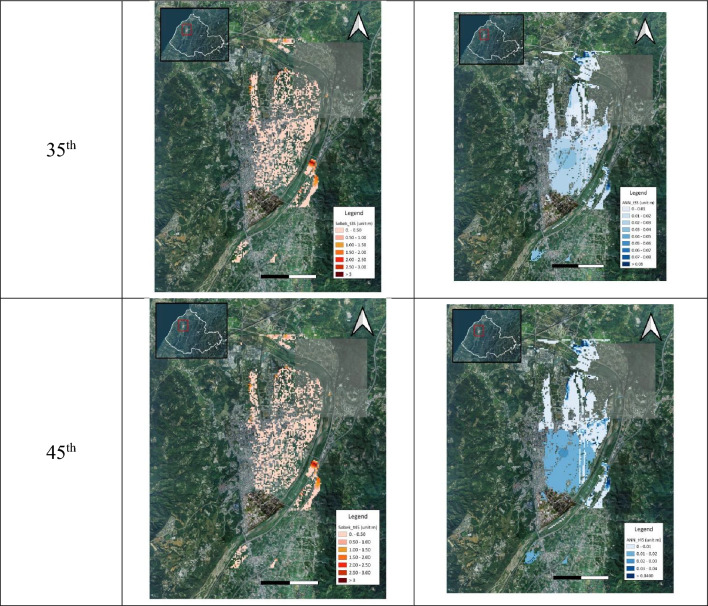
Comparison of the flooding area with the validated data at the specific time steps.

**Figure 18:**
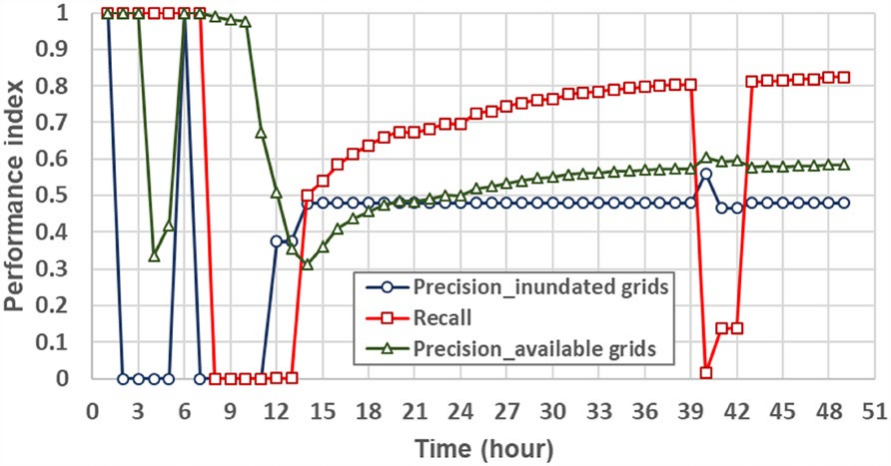
Time series of the performance indices for the evaluation of the spatial inundation distribution.

In addition to the evaluation regarding the estimations of the gridded inundation depths and flooding extent, the accuracy and reliability of the proposed SM_EID_2D model in the emulation of the flooding zones could be demonstrated by comparing the spatial distribution of the inundated grids with the nonzero simulated inundation depths via the proposed SM_EID_2D model and SOBEK model, respectively. In this study, the schematic comparison between the flooding zones at the various time steps during the validation event (see Fig. 17) could also be achieved by calculating the three model performance indices (${\theta }_{1}, {\theta }_{2} and {\theta }_{3}$) through Eqs. (17)–(19), as shown in Fig. 18 and listed in Table 7. Referring to Fig. 18, it could be observed that the results from the graphical comparison of the spatial change in the inundation distribution comprised of the inundated grids identified both via the proposed SM_EID_2D and SOBEK reply on the time steps; furthermore, most of the simulated inundated grids by the proposed SM_EID_2D model are involved in the resulting inundation spatial distribution from the SOBEK model, especially at the 15th hour–24th hour with high rainfall intensities (on average 92 mm/hour), revealing that the proposed SM_EID_2D model has an excellent ability to provide the realistic spatial distribution of inundation emulated by the SOBEK model.

**Table 7:** Statistical properties of the performance indices for the evaluation of the spatial distributions of inundated and non-inundated grids.

Validation event		Precision of inundated grids ${\theta }_{1}$	Recall ${\theta }_{2}$	Precision of the available grids ${\theta }_{3}$
Gridded inundation depth	Mean	0.410	0.644	0.522
	Standard deviation	0.224	0.316	0.283
Maximum inundation depth		0.898	0.835	0.619

1. Precision index for simulating inundated grids ${\theta }_{1}$

Figure 18 shows the results from the quantification of the above difference in the graphical comparison regarding the inundation distribution in space, indicating that the performance indices (${\theta }_{1},{\theta }_{2} and {\theta }_{3}$) have a marked increase with time, excluding the 1st time step to the 9th hours. In detail, as for the precision index ${\theta }_{1}$ subject to the accuracy of the inundated grids both by the proposed SM_EID_2D and SOBEK models, it ranges from 0.3 from the 12th time step and consistently reaches 0.5 at the end with a high mean and low standard of 0.4 and 0.2, respectively; also, according to the results from the gridded maximum inundation depth, the performance index ${\theta }_{1},$ reaches 0.9, implying that the proposed SM_EID_2D model could produce the inundated grids which is also regarded as inundated ones simulated by the SOBEK model with a half accuracy.

(2) Recall index $${\theta }_{2}$$

Moreover, the performance index ${\theta }_{2}$ mainly quantifies the performance of reproducing the spatial distribution of the inundated grids identified by the SOBEK model. Thus, given Fig. 18, the performance index ${\theta }_{2}$ reaches 1.0 at the first eight hours and gradually increases from 0.5 at the 12th hour; finally, it rises to 0.8. Even for the result from the gridded maximum inundation depths, the performance index ${\theta }_{2}$ approaches 0.8. The above implies that the inundated grids specified by the SOBEK model are more likely to be inundated ones achieved by the proposed SM_EID_2D model. The flooding zones comprised of the inundated girds identified via the proposed SM_EID_2D model could produce realistic results from the validated data under the same conditions of the heavy rainfalls.

(3) Precision index for simulating available grids ${\theta }_{3}$

Nevertheless, the above performance indices (${\theta }_{1}$ and ${\theta }_{2}$) mainly focus on the accuracy and reliability of simulating the inundated grids without considering the likelihood of achieving the non-inundated grids with the simulated inundation depths of 0 m. The purpose of the 2D inundation simulation due to a rainstorm is not only to achieve the simulated inundation depths to make concern the induced flooding regions, but also to ensure the non-flood regions comprised of the resulting non-inundated grids. In this study, the non-flood areas could be evaluated based on the performance index ${\theta }_{3}$, quantifying the precision for estimating the locations of inundated and non-inundated grids); thus, according to Fig. 18 and Table 7, the performance index ${\theta }_{3}$ stays at a constant of 1.0 between the 1st and 12th hour with non-rainfall, implying that the proposed SM_EID_2D model could precisely recognize the non-inundated grids under the condition of non-rainfall periods; the performance index ${\theta }_{3}$ then drops to 0.3 at the 14th hour and increases to a constant (about 0.6) at the end with a high value of 0.62 based on the maximum gridded inundation depths, which are significantly superior to the performance indices ${\theta }_{1}$ and ${\theta }_{2}$; this reveals that although the proposed SM_EID_2D model probably slightly overestimate the number of the inundated grids as compared to the results from the SOBEK model, it could capture the spatial distribution of non-inundated grids with higher accuracy. As a result, the proposed SM_EID_2D model could accurately delineate the flooding maps comprised of all available grids, including the inundated and non-inundated available grids, with high reliability.

## Summary

Overall, the proposed SM_EID_2D could produce more accurate and realistic inundation-depth estimates at the IoT sensors under the conditions of the rainfall observations and forecast as well as the inundation-depth observations; the corresponding inundation-depth estimates at the ungauged locations are also able to be achieved, which could persist the spatial correlation with the gauged inundation depths with an acceptable accuracy; also, the non-inundated grids linked to the zero estimated inundation depths could be accurately recognized via the proposed SM_EID_2D mode. Also, within the proposed SM_EID_2D model, the rainfall-induced simulation at the time step of 1 h, on average, merely takes 0.05 s; thus, the 1-h 2D inundation at all grids (around 6823) in the study nearly needs 5 min. On the contrary, the SOBEK model takes at least 15 min to carry out the 2D inundation simulation. As a result, the computation efficiency of the proposed SM_EID_2D model is significantly superior to the SBOEK model.

Furthermore, within the proposed SM_EID_2D model, the 2D inundation simulation is mainly carried out via the gridded ANN-derived models; accordingly, during a rainfall-induced flood process, the subbasin-based 2D inundation simulation could immediately proceed with gathering the resulting inundation depths at the particular grids from the proposed SM_EID_2D model without re-configuring the structure of the hydrodynamic numerical model for the specific zones in advance.

## Conclusion

This study aims to develop a smart model for carrying out 2D inundation simulation by estimating the gridded inundation depths with the rainfall estimations and forecasts as well as the water-level observation at the IoT sensors, named SM_EID_2D model, in which the available grids with and without IoT sensors are defined as the IOT-based and VIOT-based grids, respectively. Within the proposed SM_EID_2D model, two relationships for estimating the inundation depths are the IOT-based and VIOT-based grids via the ANN-derived ANN_GA-SA_MTF model^36^. To proceed with the model development and validation of the proposed SM_EID_2D model, 50 gridded historical rainstorms in Miaoli City are adopted in 1000 simulations of the rainfall-induced flood events as the training datasets. The results from the model validation based on a simulated flood event of 51 h indicate that the proposed SM_EID_2D model has a good ability to emulate the inundation depths at the available grids under a high accuracy reasonably in response to the temporal and spatial change in the rainfall. In addition, the proposed SM_EID_2D model is proven to efficiently carry out the 2D inundation simulation by reasonably and realistically emulating the spatial distribution of the inundated and non-inundated grids combined as the flooding map in the study area.

Although the observed inundation depths at the IoT sensors during the rainstorms are considered as the model inputs, the other hydrological variates (e.g., the infiltration, antecedent moisture status, and soil texture) which might make a significant contribution to the estimation of surface runoff and water level, should be considered to boost the accuracy and reliability of the proposed SM_EID_2D model in estimating the gridded inundation depths at the IoT sensors. Additionally, the gridded inundation depth should be impacted due to the uncertainties in the hydraulic and topographical factors, such as roughness coefficient, the DEM resolution, and slope^10,51,52^; accordingly, the ANN-derived function relationship for estimating the ungauged inundation depths would be modified with considering the uncertainty factors. Also, despite the proposed SM_EID_2D model could efficiently provide more reliable and realistic 2D inundation simulation, its computation time should reply on the number of available grids of interest; by so doing, to enhance the model computation efficiency, a task-based parallel algorithm^53^ would be coupled with the proposed SM_EID_2D model to achieve a considerable number of the estimated inundation depths at the specific grids linked to the potentially-inundated watersheds.

## Data Availability

The datasets used and/or analyzed during the current study are available from the corresponding author upon reasonable request.
